# Adsorbed Oxygen Ions and Oxygen Vacancies: Their Concentration and Distribution in Metal Oxide Chemical Sensors and Influencing Role in Sensitivity and Sensing Mechanisms

**DOI:** 10.3390/s23010029

**Published:** 2022-12-20

**Authors:** Engin Ciftyurek, Zheshen Li, Klaus Schierbaum

**Affiliations:** 1 Department of Materials Science, Institute for Experimental Condensed Matter Physics, Heinrich Heine University of Düsseldorf, 40225 Düsseldorf, Germany; 2 ASTRID2 Synchrotron Light Source, ISA, Centre for Storage Ring Facilities, Department of Physics and Astronomy, Aarhus University, Ny Munkegade 120, 8000C Aarhus, Denmark

**Keywords:** gas sensor, adsorbed oxygen, tungsten oxide, XPS, UPS, XPEEM, sensing mechanism, H_2_, metal oxides, synchrotron, characterization techniques

## Abstract

Oxidation reactions on semiconducting metal oxide (SMOs) surfaces have been extensively worked on in catalysis, fuel cells, and sensors. SMOs engage powerfully in energy-related applications such as batteries, supercapacitors, solid oxide fuel cells (SOFCs), and sensors. A deep understanding of SMO surface and oxygen interactions and defect engineering has become significant because all of the above-mentioned applications are based on the adsorption/absorption and consumption/transportation of adsorbed (physisorbed-chemisorbed) oxygen. More understanding of adsorbed oxygen and oxygen vacancies ($V_{O}^{•},V_{O}^{••}$) is needed, as the former is the vital requirement for sensing chemical reactions, while the latter facilitates the replenishment of adsorbed oxygen ions on the surface. We determined the relation between sensor response (sensitivity) and the amounts of adsorbed oxygen ions ($O_{2\left(\mathrm{ads}\right)}^{-},{\;O}_{\left(\mathrm{ads}\right),\;}^{-}O_{2\left(\mathrm{ads}\right)}^{2-},{\;O}_{\left(\mathrm{ads}\right)}^{2-}$), water/hydroxide groups (H_2_O/${\mathrm{OH}}^{-}$), oxygen vacancies ($V_{O}^{•},{\;V}_{O}^{••}$), and ordinary lattice oxygen ions ($O_{\mathrm{lattice}}^{2-}$) as a function of temperature. During hydrogen (H_2_) testing, the different oxidation states (W^6+^, W^5+^, and W^4+^) of WO_3_ were quantified and correlated with oxygen vacancy formation ($V_{O}^{•},{\;V}_{O}^{••}$). We used a combined application of XPS, UPS, XPEEM-LEEM, and chemical, electrical, and sensory analysis for H_2_ sensing. The sensor response was extraordinarily high: 424 against H_2_ at a temperature of 250 °C was recorded and explained on the basis of defect engineering, including oxygen vacancies and chemisorbed oxygen ions and surface stoichiometry of WO_3_. We established a correlation between the H_2_ sensing mechanism of WO_3_, sensor signal magnitude, the amount of adsorbed oxygen ions, and sensor testing temperature. This paper also provides a review of the detection, quantification, and identification of different adsorbed oxygen species. The different surface and bulk-sensitive characterization techniques relevant to analyzing the SMOs-based sensor are tabulated, providing the sensor designer with the chemical, physical, and electronic information extracted from each technique.

## 1. Introduction

The international gas sensor market size will reach USD 4.49 billion by 2028. The demand for semiconducting metal oxide (SMO)-based gas sensors is growing as a consequence of the sensing capabilities of these sensors to detect poisonous emissions and their adaptability for wearable/embedded designs for human health/environmental monitoring. The increasing number of Smart-City projects to combat air pollution requires air quality monitoring. Internet of Things (IoT)-compatible, wireless, SMO-based gas sensors suit the role most acceptable for environmental monitoring. Demand increased for gas sensors in the healthcare industry during the COVID-19 pandemic for assessing the levels of oxygen (O_2_), carbon monoxide (CO), carbon dioxide (CO_2_), nitrogen dioxide (NO_2_), and other gases that contribute favorably to monitoring human health and wound healing for the elderly; these gases are much needed. The published research on gas sensors was 3696 papers in 1999, which increased after a decade to 8797 in 2009. After another decade, the volume of the papers published in just 2019 reached to 23,506 and drastically rose to 33,452 toward the end of 2022. These numbers was retrieved from ScienceDirect. The outcome of these research activities brought a great deal of understanding and led to developments in these fields.

A proper definition for a chemical gas sensor output is “analytically useful signal results from a chemical interaction between the target gas and the sensor surface, converts chemical information of a quantitative or qualitative sort”. In particular, semiconducting metal oxide (SMO) sensors are the most studied and widely produced technology. The working principle of this type of sensor is based on the alteration of the electrical resistance of the metal oxide semiconductor when it is exposed to the target gases. Such chemical gas sensor devices are called different names such as chemiresistors or resistive chemical sensors or chemiresistive sensors. SMO-based sensors suffer primarily from a lack of gas selectivity, which impedes the development of low-cost and reliable systems for gas monitoring. Target gases react with chemisorbed oxygen. Consequently, SMO-based sensors cannot distinguish target gases based on a simple sensing mechanism. The most common metal oxides ZnO, SnO_2_, MoO_3_, TiO_2_, and WO_3_ are sensitive to many different gases, including but not limited to H_2_, NO/NO_2_, H_2_S, CH_4_, O_2_, CO, CO_2_, SO_2_. The active surface structure of SMOs boosts their applications as gas sensors widely but brings a significant weakness: selectivity [1,2,3]. Due to the above-given reasons, it is critically important to understand the precise working conditions of the SMOs under realistic/near-realistic working conditions, employing in situ analysis during exposure to the most common and practically important target gases, such as hydrogen (H_2_). The increasing need for monitoring and impeding harmful emissions is a central aim of stopping climate change. There is a significant need to develop optimized H_2_ and CO sensors. The high demand for H_2_ gas sensors is not limited to industrial process control and leak detection applications and extends to the food and medical industry, such as diagnosing gastrointestinal diseases (GIDs). Hydrogen has the prospect of becoming a unique energy source due to its direct exhaustible nature. The detection of low concentrations as well as at the 4% explosive limit of H_2_ is required because of its small size and diffusion through almost everything, low ignition energy, and wide explosive concentration range during transportation and/or storage and use; in this way, the final consumer trust in H_2_-based fuel technologies can be established [4,5].

**Figure 1:**
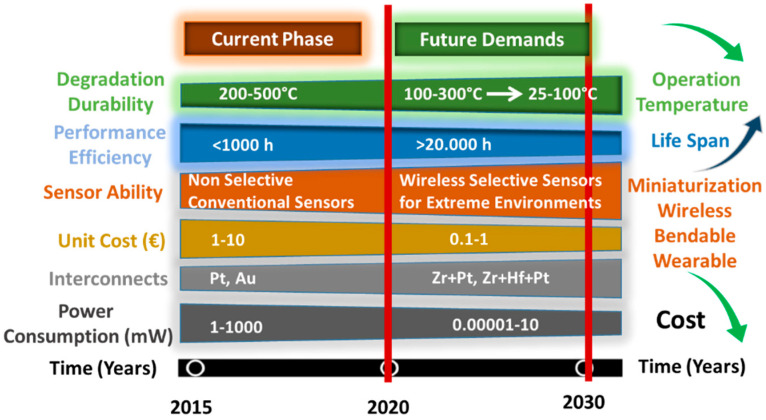
A 2030 roadmap for development of gas sensors.

Figure 1 represents the future projection regarding the current state-of-the-art chemical gas sensor development in the scope of the desired developments in the era of 2020–2030. As can be seen from the figure, the main targets of the time as of 2022 are the development of high sensitivity-selectivity gas sensors with low cost, improved stability, online monitoring wireless communication capabilities, and bendable-wearable SMO designs for the healthcare industry. The above-listed development targets encompass alignment with the IoT and the connection of humans with the rest of the environment [6]. The targets for unit cost reduction will benefit from the utilization of inexpensive interdigitated electrodes such as gold (Au) or platinum (Pt) being partially/entirely replaced with zirconium (Zr) and hafnium (Hf) [7,8]. A decrease in operation temperature brings both high durability and lower power consumption. Miniaturization will convey the advantage of low power consumption and the integration of multiple sensing architectures on a single platform, as with one such example used in this current work. The increased selectivity will bring an unprecedented understanding of processes, quality of life, and environmental protection. The current phase for scientists and engineers is evolving from non-selective sensors to selective, multipurpose, low-cost, low-energy-consuming sensing technology. Scientists and engineers struggle to address miniaturization, selectivity-sensitivity prospects, and high demands from the consumer market. The robustness and compatibility with silicon processing make the semiconducting metal oxide (SMOs) sensors the top candidate to meet the market demands. Bringing low power consumption together with selectivity and sensitivity remains a significant challenge. In order to increase the sensitivity-selectivity of a gas sensor, designers need to clarify the actual type of adsorbed species and their dynamic behavior in the course of the chemical reactions with target gases. Gas-sensing mechanism explanations will also benefit from the kinetics and equilibria of oxygen adsorption, and the identification of the amount of adsorbed species present at the sensor surface as a function of temperature.

Despite massive research applied to SMOs, there are many unknowns to the SMOs themselves, especially under gas sensor operation conditions. Surprisingly, it is not even clear the basis of n-type conductivity in some of the valuable SMOs such as ZnO and In_2_O_3_. For WO_3_, the origin of the donor levels in the electronic band-gap has been debated for a long time. [9,10]. Mainly, researchers paid attention to the nanostructured SMOs due to their large surface-to-volume ratio leading to high surface activity. Very few papers focused on the origin of that high surface activity. The surface defects such as oxygen vacancies ($V_{O}^{•},{\;V}_{O}^{••}$) are game-changers in the surface catalytic activity and electrical resistance. Observing the surface status and defect dynamics under in situ conditions similar to the gas sensor operation conditions is required. More importantly, it is essential to measure the chemisorbed oxygen species. Adsorbed oxygen ions dictate the sensing mechanism/reactions in SMO-based sensors; thus, comprehending them will bring an understanding of the sensing mechanisms involved together with developing high-selectivity and sensitivity sensors.

In order to address these challenges, the surface approach is used in this work. This approach encompasses analyzing the conditions for the gas sensor works and measuring the surface adsorbed oxygen ions ($O_{2\left(\mathrm{ads}\right)}^{-},{\;O}_{\left(\mathrm{ads}\right),\;}^{-}O_{2\left(\mathrm{ads}\right)}^{2-},{\;O}_{\left(\mathrm{ads}\right)}^{2-}$), water/hydroxide groups (H_2_O/${\mathrm{OH}}^{-}$), oxygen vacancies ($V_{O}^{•},{\;V}_{O}^{••}$), and ordinary lattice oxygen ions ($O_{\mathrm{lattice}}^{2-}$), together with different oxidation states (W^6+^, W^5+^, W^4+^) found in WO_3_ during gas sensor testing. The final step is establishing a correlation with the sensor response for the highest sensitivity. The current work establishes the surface morphology, stoichiometry, valance band, surface defects, and the amount of adsorbed oxygen ions as a function of temperatures ranging from 25–400 °C.

## 2. Overview of Adsorbed Oxygen from the Viewpoint of Gas Sensor Designer

The present section aims to provide an overview of the findings on adsorbed oxygen ions and chemisorbed oxygen species and their role in semiconductor metal oxide (SMO)-based gas sensors’ functionality. Chemiresistive-type gas sensors operate based on reduction-oxidation (Redox) reactions on the SMOs’ sensing material surface upon exposure to the target gas. The basis of those reactions is the depletion of adsorbed oxygen ions in an n-type SMO (such as WO_3_, TiO_2_, SnO_2_) for reducing gases (such as H_2_, CO, SO_2_, H_2_S), while the opposite is the augmentation of oxygen ions promoted on the SMOs’ surfaces for oxidizing gases (such as NO_2_/NO, O_3_, O_2_). Any explanation of the chemical sensing mechanism should derive input from the surface stoichiometry of SMO, equilibria of oxygen chemisorption, and the identification and the amount of adsorbed species, including oxygen, water, and hydroxides present at the sensor surface as a function of temperature. The behavior of these oxygen-containing species in the gas-sensor-related reactions, such as oxidation or reduction, depends on the target gas composition, which will further strengthen the understanding of the gas sensing mechanisms involved. The forms of adsorbed oxygen species are typically acknowledged as electrically neutral molecular oxygen (O_2_) and negatively charged chemisorbed oxygen ions ($O_{2\left(\mathrm{ads}\right)}^{-},{\;O}_{\left(\mathrm{ads}\right),\;}^{-}O_{2\left(\mathrm{ads}\right)}^{2-},{\;O}_{\left(\mathrm{ads}\right)}^{2-}$), as the former exists at a physisorbed state in the room temperature range while the latter chemisorbed ion forms can sustain up to 400–500 °C. Multiple layers of oxygen-containing adsorbed species cover the surface of SMOs. These compounds are chemisorbed oxygen ions and hydroxides, and the whole layer is covered by atmospheric, post-depositional, and/or deposition-related carbon-oxygen compounds. These oxygen species and hydroxides are the key factors determining the interaction of the SMO with the target gases.

Historically, chemisorbed oxygen ions have been the subject of scientific interest in catalysis and corrosion science dating back to the 1960s for the interpretation of native oxide formation on metal surfaces [11,12,13,14], thus focusing on chemisorbed oxygen and precious metal interactions, such as platinum (Pt) and silver (Ag), by means of electrochemical and electrical measurements [15]. Many experimental and theoretical research works are available to explore the interaction of H_2_, H_2_S, SO_2_, H_2_O, NO_x_, CO, O_2_, and CO_2_ with catalytically active metals such as Cu, Pt, Pd, Ru, Ag, Au, and Ni surfaces [16,17]. MacDougall and Cohn developed a model clarifying nickel (Ni) oxidation at room temperature with electrical measurements without having an atomistic view of the surface due to limitations in the then-available surface-sensitive spectra-microscopic techniques. The authors deduced that nickel oxide (NiO) construction starts with oxygen chemisorption, reaching a surface coverage of a monolayer of chemisorption followed by lateral growth, then turning into inward growing oxide. The authors did not elaborate on pinpointing the active chemisorption sites, the type of the chemisorbed oxygen species, and their stability [18]. A few papers from catalyst-corrosion-related disciplines focused on metal-oxygen surface interactions with in situ methodology with diverging research interests concerning a chemical sensor design. The methods used to characterize those interactions and products cover many analytic/spectroscopic/microscopic techniques. While this research has provided the way to a basic understanding of the dissociation and adsorption properties of different forms of oxygen ions, it did not provide an understanding of the chemical sensor design, especially working with metal oxides rather than pure metals.

Physisorbed and chemisorbed oxygen may react with H_2_ to form H_2_O. Physisorbed species are bonded to the solid by van der Waals forces, so their removal will not impact the distribution of electrical charge in the solid. However, chemisorbed oxygen ions can extract electrons, decreasing electrical conductivity on the surface. There are various forms of oxygen species on the surface at room temperature. Those that are subjects of interest for chemical sensor designers are negatively charged oxygen species such as ($O_{2\left(\mathrm{ads}\right)}^{-},{\;O}_{2\left(\mathrm{ads}\right)}^{2-},\;O_{\left(\mathrm{ads}\right)\;}^{-},{\;O}_{\left(\mathrm{ads}\right)}^{2-}$). Some of the chemisorbed oxygen ions can sustain on the SMO’s surface at up to 400–500 °C, while 100 °C is the maximum temperature at which molecular oxygen (O_2_) can stay. Chemisorbed oxygen ions also influence molecular oxygen adsorption, and the concentration increase adversely affects the adsorption of molecular oxygen [19]. At room temperature, molecular adsorption of oxygen can be depicted as a reversible process, while chemisorption is not. Another meaningful difference in these processes is that molecular adsorption of oxygen does not involve a change in electrical properties or the work function (Φ); conversely, chemisorption affects both significantly.

**Figure 2:**
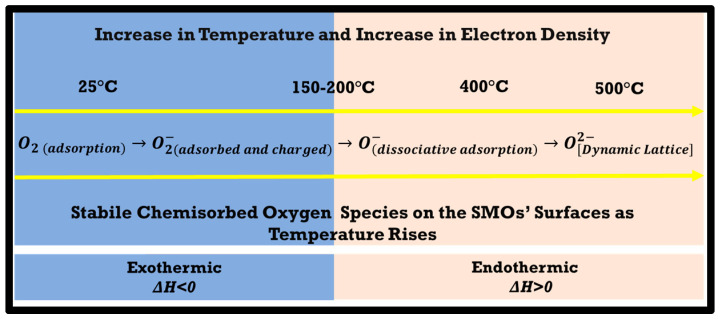
Transformation and stability of oxygen ions as temperature rises on SMOs’ surfaces.

Adsorbed oxygen ions are essential for catalytic oxidation processes occurring above room temperature. Figure 2 shows the scheme for transforming oxygen species as temperature rises, from physisorbed to chemisorbed, then to lattice incorporation. Electron concentration in individual oxygen ions increases from left to right. It is a matter of discussion about the stability of surface oxygen species on the SMO surface. Among other likely intermediate ionic forms of oxygen, only the superoxide ion ($O_{2\left(\mathrm{ads}\right)}^{-}$) is stable compared to the gaseous O_2_. Therefore, the $O_{2\left(\mathrm{ads}\right)}^{-}$ is the most commonly reported adsorbed oxygen species. All other species are unstable in the gas phase. As the temperature rises, $O_{2\left(\mathrm{ads}\right)}^{-}{\;\mathrm{to}\;O}_{\left(\mathrm{ads}\right)\;}^{-}$ transformation occurs, and at around ~150–200 °C, the diatomic oxygen ion either leaves the surface or dissociates further into $O^{-}$. At the same time, monoatomic oxygen ions can stand up to temperatures >400 °C, depending on the sample surface, defect density, type of metal, testing environment, and oxygen partial pressure [20,21]. $O^{2-}$ ions are long-lasting on a SMO’s surface, especially at elevated temperatures, unless not trapped by oxygen vacancy sites ($V_{o}^{•}$ and $V_{o}^{••}$). $O^{2-}$ is in its firmest form as incorporated into a lattice configuration by being perfectly balanced in crystal symmetry. It is mainly agreed that $O_{2}^{-}$ and $O^{-}$ are also stable chemisorbed oxygen species [19]. The formation of $O_{2}^{-}$ results in a decrease in the free energy around ~1 eV, while all other transformations are endothermic reactions. The dissociation of the $O_{2}^{-}$ to $O^{-}$ requires ~0.5 eV, the formation of $O_{2\left(\mathrm{ads}\right)}^{2-}$ further requires ~5 eV, and a formation of $O^{2-}$ will need ~20 eV. All reactions are facilitated by the SMOs’ surfaces, as the given energies will vary from metal oxide to metal oxide and will also vary as a function of the surface stoichiometry/chemistry of SMOs.

The most enduring form for the adsorption of oxygen by the SMOs is its consolidation into the lattice in the form of an $O^{2-}$ ion, while its dynamic balance ($O_{\left[\mathrm{Dynamic}\;\mathrm{Lattice}\right]}^{2-}$) will bring functionality to the gas sensor at elevated temperatures, >500 °C. Figure 2 does not necessarily show the thermodynamic energy decrease of the system as well as the stability of the individual ions in the gas phase. In contrast, it shows the stability of the adsorbed ions on the SMOs’ surfaces as temperature rises from left to right. For nickel oxide (NiO), $O_{2\left(\mathrm{ads}\right)}/O_{2\left(\mathrm{ads}\right)}^{-}{\;\mathrm{to}\;O}_{\left(\mathrm{ads}\right)}^{-}$ transformation peaks around 150 °C and then slows down; however, beyond ~235 °C, no data were presented. Another study concluded that oxygen could be adsorbed to a platinum (Pt) surface up to 360 °C, peaking at around 300 °C under 10^−7^ mbar of O_2_ partial pressure for an hour [22]. Jones et al. found that oxygen adsorption on silver (Ag) metal starts at 150 °C and transforms to the chemisorbed state [23]. Tan et al. reported room temperature oxidation of aluminum (Al) thin film in lower stoichiometry (Al_x_O_y_), as x/y is almost equal to ~1 whose surface is covered with chemisorbed oxygen ions [24]. Depending on the operational temperature, one or more of these adsorbed oxygen species will be in a dynamic equilibrium with the environment. The presence of $O^{2-}$ in the dynamic adsorption-desorption process is observed up to 500 °C [21,25,26,27]. Above 500 °C, it is reported that there are multiple dynamic equilibriums under certain specific oxygen partial pressures. SMO vapor pressure facilitates dynamic balance in favor of oxygen chemisorption, eventually ending in lattice transformation and interstitial oxygen supportive pumps up to 600 °C, even up to 925 °C for specific cases [1,2,28,29].

Deriving interpretation over different types of chemisorbed oxygen species possesses a great deal of difficulty. Most of the early available literature focuses on the effects of adsorbed oxygen on the electrical conductance, fluorescence, photo conductance, and catalytic behaviors of ZnO and TiO_2_, not explicitly focusing on the sensing mechanism explanations. Paramagnetic $O_{2}$, $O_{2}^{-}$, and $O^{-}$ ions can be distinguished by EPR analysis [30,31]. The other species such as $O_{3}^{-}$ and $O_{4}^{-}$ can be detected at liquid nitrogen temperature with maximum care and do not really exist in the near or full functionality conditions for chemical sensors. EPR findings showed that $O_{2}^{-}$ and $O^{-}$ dominate ions at low and high temperatures, respectively, as the transition temperature between the two regimes is ~150–200°C. At the same time, it should be noted that EPR lacks a signal for $O^{2-}$ ions and also oxygen ions found in diamagnetic H_2_O [32], while $O^{2-}$ is one of the most active adsorbed oxygen ions capable of existing at higher temperatures.

Since the initial work on titanium by Simmons et al. [33], a few investigations identified the surface concentration of hydroxide groups (${\mathrm{OH}}^{-}$) on oxide-covered metals by using photoelectron-based methods (XPS-UPS). Dang and Kurbatov quantified the concentration of ${\mathrm{OH}}^{-}$ groups at the oxide-covered iron surface [34,35]. McCafferty and Wightman [36] took a step further and applied Simmons’ approach for aluminum (Al), chromium (Cr), tantalum (Ta), and silicon (Si) to calculate the hydroxide (${\mathrm{OH}}^{-}$) to lattice oxygen ratio. Chemisorbed oxygen ions were not included. In several instances, XPS-UPS were used to understand the existence of chemisorbed oxygen ions. One of the earliest works came from Bateau and Madix in a series of papers [22,37,38]. The authors utilized UPS for analysis of adsorption and interaction of CO, $O_{2}$, and $H_{2}O$ on single crystalline precious metals. Authors hypostasized that island-like structures are hydroxide (${\mathrm{OH}}^{-})$ with additional evidence from mass spectroscopy (MS), thermal desorption (TPD), LEED, and HREELS for the structural interpretation. The authors did not elaborate on the amount of chemisorbed oxygen ions among other oxygen-containing species. In other studies, authors applied low-pressure levels of CO, H_2_, and O_2_ to understand the surface chemistry and work function (Φ) changes. O 1s and valance band spectrums were analyzed; while experimental conditions were perfect for the chemisorption of oxygen, authors excluded chemisorbed oxygen ions by indicating that O 1s can be divided into three main components: ${\mathrm{OH}}^{-}$, H_2_O, and $O_{\mathrm{Lattice}}^{2-}$. Other researchers identified chemisorbed oxygen ions as oxygen vacancy ($V_{O}^{••}$)-related regions, hydroxide/water groups (H_2_O, ${\mathrm{OH}}^{-}$), and/or bulk hydroxides, e.g., SnO_x_H_y_, or sometimes CO, CO_2_, (${\mathrm{CO}}_{3}^{2-}$)-related carbon- and oxygen-containing groups.

Chemisorbed oxygen ions ($O_{2\left(\mathrm{ads}\right)}^{-},{\;O}_{2\left(\mathrm{ads}\right)}^{2-},\;O_{\left(\mathrm{ads}\right)\;}^{-},{\;O}_{\left(\mathrm{ads}\right)}^{2-}$) are hard to distinguish from ${\mathrm{OH}}^{-}$ and H_2_O and other oxygen-containing adsorbed species (such as CO, CO_2_, ${\mathrm{CO}}_{3}^{2-}$) due to the proximity of their binding energies in XPS-UPS measurements. The proximity of the oxygen-containing species (water, hydroxides, chemisorbed, physisorbed, organic contaminations) to each other is ~0.5–1.0 eV [25,33,39,40] in the electron binding energy (BE) scale for SMOs. Limitations in the measurement systems, X-ray photon sources, electron analyzers, and, more importantly, a narrow interest in understanding chemisorbed oxygen ions lead to the inability to distinguish them from other functional surface groups. Although having proper conditions for the existence of chemisorbed oxygen ions on metal oxides (SMOs), the occurrence of them has not been regularly reported, and chemisorbed oxygen species have been accounted for in oxygen vacancy-related sites ($V_{O}^{••}$), interstitials oxygen ions ($O_{i}^{\prime \prime }$), adsorbed water (H_2_O), and hydroxide species (${\mathrm{OH}}^{-}$) for many different material sets [41,42,43,44,45,46,47,48,49,50]. Another layer of complexity arises from the defective nature of the SMOs’ surfaces, surface roughness, and sub-surface crystal potentials, leading to a considerable variation in binding energies (BEs) reported for O 1s. Those reasons lead to the chemisorbed oxygen species being mistaken as the above-mentioned surface functional groups.

Clifford and Windischmann determined that bulk electrical conductance results from oxygen vacancies ($V_{O}^{••}$,$V_{O}^{••}$). The authors also indicated that the oxygen vacancy concentration is the rate-limiting step of oxygen adsorption [51,52,53]. The researchers showed that even highly stabile oxide Al_2_O_3_ in its stoichiometric form develops chemisorbed oxygen ions bound to the surface by positively charged oxygen vacancies by employing relatively high-level O_2_ doses via ex situ XPS investigations [54,55,56,57]. A few studies from catalysis research combined their XPS work with qualitative understanding regarding the orientation of adsorbed oxygen ions derived from XANES on metallic substrates such as Ag with pressures far lower than actual operating conditions for gas sensors [23,56]. XANES and EXAFS techniques were also utilized to gain information on the sensing mechanism of La_2_O_2_CO_3_-based gas sensors upon exposure to CO_2_. [58]. Studying the adsorbed oxygen ions with these bulk confined techniques was very difficult. Moreover, these techniques lack quantification methods and guidelines; however, proper quantification guidelines and practices are very detailed, precise, and straightforward in XPS-UPS analysis.

## 3. Review of Analytical-Spectral-Microscopic Tools for Semiconducting Metal Oxides (SMOs), Adsorbed-Chemisorbed Oxygen, and Chemical Gas Sensors Analysis

SMO-based chemical sensors can be characterized in regards to their non-stoichiometry in their metal-oxygen sublattice, and such can be found in WO_3-x_ or SnO_2-y_, determined by the magnitude of ‘*x*’ and ‘*y*’. Other aspects of sensor analysis include but are not limited to elemental analysis, electronic-electrical properties, surface topography, and crystallinity. The non-stoichiometric nature of SMOs brings the semiconducting effects and desired surface properties, such as dissociation sites for oxygen and target gas. Thus, it is vital to characterize oxygen stoichiometry on the surface. Chemical sensor researchers mainly collect the required analytical-spectral-microscopic tools, employing the techniques given in the succeeding paragraphs and detailed in Table 1 [21,26,59,60,61,62,63,64,65,66,67,68,69,70,71,72,73,74,75]. The full names of each technique detailed in Table 1 is given in the in Abbreviations section at the end of the paper.

Table 1 details the surface and bulk sensitivity of the specified spectroscopic, chemical, analytical, and electronic information of the techniques named above that can provide practical, helpful, and application- and design-related basic and advanced information for chemical sensor designers. At the same time, the succeeding paragraphs provide critical insights concerning every technique listed. It is challenging to determine the amount, distribution, and compositional variations of oxygen-containing species on the chemical sensor SMOs’ surfaces. This challenge is due to: (i) requirements of surface-sensitive techniques, (ii) difficult experimental conditions to realize the sensor operation environments, and (iii) physical and analytic difficulties in identifying and distinguishing chemisorbed oxygen species from hydroxide/hydroxide and other oxygen-containing functional groups. Some of the techniques listed in Table 1 are surface-sensitive and in situ/operando applicable, thus capable of detecting adsorbed surface species. In contrast, the others in the list provide bulk information that dominates the measured data by imposing high suppression on the analytically significant surface adsorbed species. The following paragraphs will review each technique above regarding the sensor-sensing material SMOs’ analysis.

Surface chemisorbed oxygen species estimated from equilibrium bulk measurements such as activation energy and EPR are under question due to uncertainty regarding the oxygen vacancies and interstitial sub-surface cations. In such techniques, there is no direct evidence for forming oxygen vacancies ($V_{O}^{••}$) for freeing electrons to the conductance band, which can either result from the formation of oxygen vacancies or interstitial inclusion of the cation into the lattice [76]. EELS is not a surface-sensitive technique, while REELS-HREELS are surface-sensitive due to electrons scattered from the surface of a studied sample. EELS data originate from measurements of differences in the energy and angular dispersal of scattered electrons during transmission through the sample of interest. In the best scenario, EELS spot analysis will provide the local chemical composition, valence states, and average nearest neighbor distances. EELS is a transmission/absorption technique; thus, the incident beam must penetrate the specimen. The polycrystalline SMOs’ sensing layers applied with conventional thin/thick film procedures are not standalone. They are thicker than ≥100 nm, so the application probabilities of EELS drastically decline for the chemical sensor applications. At the same time, both techniques suffer from difficulties in the quantification and availability of the standards [77]. RBS utilizes high-energy He or H ions (1.0–3.4 MeV energies), in contrast to ISS (100 eV–5 keV). RBS-ISS are elemental quantitative depth-profiling techniques, as RBS is for bulk while ISS is for surface analysis. An elegant example is the work of Cox et al.: the authors calculated a metal-to-oxygen ratio of the SnO_2_ at the surface (110). ISS and other techniques were utilized to show selective isotopic labeling (with 18 O) of different sites to explore which surface oxygens participated in surface reactions. It also brings the advantage of quantifying the amount of H, which is impossible with XPS. The chemical status analysis is not possible with both RBS and ISS [78,79,80].

XASF spectrum can be categorized into two primary segments: EXAFS and NEXAFS/XANES. A XASF spectrum is formed by measuring the yield of X-ray emissions due to inner shell transfer upon exposure to the high-energy X-ray photons with varying energies. Those emitted X-rays are not directly providing analytic information concerning the chemical bonding of the ions. Conversely, X-ray-induced electron emission techniques such as XPS are powerful in providing chemical bonding information because the source of information is entrenched in electrons’ origination from the ions in question [81]. EXAFS is a probe of the interatomic distances, numbers of neighboring atoms (octahedral, tetrahedral, etc.), and degree of disorder in the vicinity ~0.5 nm of the X-ray-absorbing ion/atom. The “near-edge” and the adsorption edge provide information about the oxidation state or, more broadly, the local charge distribution in molecular functional groups found in polymers. The surface sensitivity of NEXAFS and EXAFS can be regulated through measurement parameters and sample selection criteria. Chemical state quantification and differentiating oxygen species through these techniques are highly challenging for a gas sensor designer. EXAFS itself is not surface-sensitive and has a high photon damage probability. SEXAFS and NEXAFS are surface-sensitive but do not provide a quantitative understanding of the chemical states of the constituent elements of the compound or the chemisorbed species; additionally, the information provided is qualitative. SEXAFS features provide structural information, nearest-neighbor bond lengths, and coordination numbers for atoms at or near the surface, while NEXAFS gives information on local coordination. The radiation damage in the XAS family techniques due to high-energy hard X-rays in the range of 3–20 keV will affect the stoichiometry of the SMOs on the surface and disintegrate, disrupt, and terminate chemisorbed oxygen species [58]. Contrary to the XPS, the XAFS may show a distinct peak formation separated by several eV belonging to interstitial oxygen ions ($O_{i}^{\prime \prime }$), which is difficult for XPS. However, XAFS is generally not considered a quantitative technique. This is based on the fact that when interpreting the XAFS results in terms of unoccupied electron states, the excitation of a core electron is followed by an electron excitation to an unoccupied state. These two-step effects, together with the core-hole influence on the unoccupied states, make it hardly an effective tool for a quantitative study on SMOs. The tandem operation of XPS and XAFS can measure the surface adsorbed species and embedded defects. One of the very few studies utilizing a combined XPS and XAFS approach focusing on surface adsorbed oxygen concluded that two types of adsorbed oxygen species were detected as 528.3 and 530.4 eV on an Ag metal surface at 125–350 °C substrate temperature [82]. Another work with combined XPS and XAFS reported three different adsorbed oxygen ions with binding energies of 530.32, 529.20, and 528.29 eV on a single-crystal Ag surface after exposure to 10^−4^ mbar O_2_ at 150 °C for 22 min. The same study also indicated that the component located at the higher binding energy side increased in intensity as the exposure time doubled [83].

Vibrational spectroscopies such as Raman, MNR, DRIFT, and IR intrinsically are not surface-sensitive techniques. Moreover, they are not capable of detecting chemisorbed oxygen species themselves. At the same time, they help analyze the adsorption kinetics of CO, H_2_O, CO_2_, and/or potentially other reducing and oxidizing gases under in situ conditions [84,85], while it is always likely that the absorption from the background gas, interstitial ions, or substrates will overwhelm the much smaller IR signal corresponding to the surface adsorbed oxygen species [86,87,88,89]. The FT-IR can be tailored as a surface-sensitive technique with the inclusion of silver (Ag) nanoparticles on the sample surface. Still, it will lose its ability to determine the original sensor surface properties due to the catalytic effect of Ag nanoparticles. Additionally, this will bring changes in the chemical environment on the surface; thus, it will lead to a misleading understanding of the surface for sensor designers [36]. Modified IR-based vibrational spectroscopic approaches, infrared reflection absorption spectroscopy (IRAS), polarization-modulation infrared reflection absorption spectroscopy (PM-IRAS), sum-frequency generation vibrational spectroscopy (SFGVS), and diffuse reflectance infrared Fourier transform spectroscopy (DRIFT) bring a certain degree of surface sensitivity, while their sensitivity for detecting minute amounts of reaction products or adsorbed species is under question [90,91]. These techniques, including IRRAS and DRIFT, are not suitable for measurements on the poly-crystalline SMOs’ bulk structures that the majority of the gas sensors are made out of. They are typically applied for single-crystalline metal oxides or oxide-thin films in single crystalline form. Several studies examined the adsorption of CO, NO, O_2_, H_2_, and CH_3_OH on mono-metallic and bi-metallic metal surfaces such as Pt, Pd, Rh, Ru, Au, Co, PdZn, AuPd, CuPt, etc. in a single-crystal or an ultrathin film form. XRD is based on the illumination of a beam of X-rays (most commonly utilizes Cu K_α_, 1.5406 Å) on a specimen. According to Bragg’s law, the XRD spectrum is established by the diffraction of the X-rays in the specimen’s crystalline phases. The intensity of the diffracted X-rays is measured as a function of the diffraction angle. The diffraction pattern is used to determine the SMO sensing materials’ crystalline phases, epitaxy, strain-stress, grain-crystalline size, and surface texture. Although this will be highly indirect, XRD can explain the SMOs’ elemental and chemical state status. XRD can also systematically analyze concentration gradients and thin/thick SMO film thicknesses. XRD can be tailored to surface sensitivity in the GI-XRD mode. EDS, EDX, or EDAX is all abbreviated to describe the same technique based on the collection and dispersion of characteristic X-ray emissions upon exposure to high-energy electrons under an ultra-high vacuum (UHV). The EDS’ most frequent chemical sensors are used to identify the sensor surface’s elemental constituents before/after gas exposure and convert them into an elemental weight concentration. EDS, almost all of the time, is found to be connected to the SEM. SEM is the first analytical technique used by the sensor designer to look at SMOs.

The photon energy in PL is 0.6–6 eV, an equivalent of 207–2070 nm in a wavelength corresponding to near-infrared, visible, and near-ultraviolet photons. A considerable number of electronic changes of interest stands in this range. XRF is a high-energy form of PL applying X-rays and is interested in core electrons instead of valence electrons, which are the interest of PL [92]. XRF and PL are used both qualitatively or semi-quantitatively to understand the correlation between the elemental composition and electronic states and determine the existence and type of impurities and defects in the data acquisition depth range of several micrometers. XRF is commonly operated for elemental quantitative analysis. XRF in total reflection mode (X-Ray fluorescence analysis (TXRF)) will bring the surface sensitivity. An X-ray beam in grazing incidence geometry will bring 1–5 nm of depth resolution. X-ray reflectivity measurements at small angles provide information about the electron density profile normal to the surface. The CL signal is induced by detecting the same photon energies used in PL (ultraviolet, visible, and near-infrared regions of the spectrum) that are emitted as the result of electron-beam bombardment, leading to electronic transitions between the conduction band, impurity- and defect-related levels in the band gap, and the valance band. CL and PL are not intrinsically surface-sensitive techniques, while CL can be adapted for surface sensitivity by varying the electron-beam energy so the excitation depth can be changed from about 10 nm to several micrometers. Both techniques may provide valuable insight, especially into oxygen vacancies ($V_{O}^{••}$) and interstitial oxygen ions ($O_{i}^{\prime \prime }$), respectively, and can be used as complementary tools for the SMO-based gas sensor surface analysis. In the UV/VIS part of the electromagnetic spectrum, electronic transitions are used to help identify unknown molecules enveloping for an electronic characterization of SMOs, such as band gap measurements. Surface sensitivity cannot be achieved intrinsically. AES is a highly surface-sensitive technique but is not comparable to the XPS in quantification capabilities. However, it is superior in qualitative analysis of the adsorbed species, especially in physisorbed or monolayer surface coverage. AES has an advantage of spatial resolution compared to XPS, while XPS has the solid upper hand concerning chemical bonding state information. EDS is not precise and lacks surface sensitivity and has a poor energy resolution. EDS is most often employed in quantitative elemental analysis rather than in the distinctions between chemical bonding and electronic structure. Therefore, a general criterion for identifying the surface species is not satisfied, while XPS can achieve such identification efficiently. UPS is an effective and appropriate technique to study the electronic structure, defect states, valence bands, and work function (Φ) of SMOs.

X-ray photon correlation spectroscopy (XPCS) is a potential future chemical sensor analysis technique. XPCS toward chemical sensors promises to detect dynamic fluctuations induced by adsorption by using resonant far-field scattering of highly coherent X-rays. Future advances in brighter synchrotron sources will bring coherent X-ray techniques like XPCS closer to the service of capturing reaction kinetics [93]. More profound knowledge of the surface properties of SMOs can be achieved by employing temperature-programmed techniques (TPD, TPO, TPR, TGA, DSC). Those techniques are employed to gain information about the thermodynamics and kinetics of adsorbed/desorbed species on surfaces, similar to those actively utilized in chemical sensing applications [2]. The reduction peak temperature indicates the ease/difficulty of reduction and the degree of interaction between gas species and surfaces, while multiple peaks indicate the presence of metal in different chemical states. Oxidation and reduction are measured by TPO-TPR, while TPD-TGA measurements will give the type and number of adsorbed species.

### The Concerns with XPS Analysis of Chemisorbed-Adsorbed Oxygen Ions on SMOs’ Surfaces

One of the best and most physically proper strategies is to use XPS to determine differences between chemisorbed oxygen from hydroxides, lattice, or hydrated compounds. The spectral resolution achievable in a synchrotron is 0.1–0.3 eV. Considering the ~1 eV difference in the lattice and chemisorbed oxygen ions’ binding energy (BE) in the XPS spectrum, the benefit of utilizing the synchrotron-based XPS appears to be progressive. At the same time, XPS binding energies determined through synchrotron-based XPS measurements can comfortably be used in the laboratory-based XSP peak deconvolution-fitting procedure. Moreover, this will lead to precise fitting for relevant binding energy values and correct amounts for the absorbed chemisorbed oxygen species. Aluminum K_α_ X-rays have a full width at half maximum (FWHM) of 0.5–0.9 eV for pure elements and 0.8–1.2 eV for some multivalent compounds relevant to gas-sensing SMOs. Spectrometer-related broadening and inherent broadening of the O 1s line in the complicated chemical environments found in polycrystalline oxide surfaces with different surface functional groups, vacancies, adsorbed phases, multiple oxidation states, and surface roughness typical of sensing SMOs will make the attempted deconvolution analysis challenging. Most XPS and UPS studies have focused on metallic thin film or foil surfaces such as Ag, Pt, and Au. In these studies, the surface properties, such as elemental identification, stoichiometry, impurity levels, and surface composition after testing with different gases, were investigated without acknowledging the existence of the chemisorbed oxygen species [49,94,95]. A few works interpreted the XPS measurements to acknowledge the contribution of chemisorbed species to surface electrical resistance and their capabilities of blending into the lattice with an increased oxygen partial pressure and temperature [96]. There have been a few attempts to quantify the chemisorbed oxygen ions through XPS. Most of the time, ion sputter cleaning (Ar, Ga, Xe energetic ions) is required due to cleaning of adventitious carbon contamination on SMOs’ surfaces [13,29,55,97]. This procedure eliminates the chemisorbed oxygen ions, as they do damage down to 50 nm from the surface [97]. SMOs are very sensitive to photon/ion-induced damage. The tungsten oxide (WO_3_) stoichiometrically reduces to the metallic state tungsten (W) after prolonged X-ray/ion exposure. It requires a high enough X-ray photon intensity to distinguish very low concentrations of chemisorbed oxygen ions among all other oxygen-containing surface groups in very short measurement times. The synchrotron X-ray source is more than four orders of magnitude higher in intensity than laboratory-based sources, leading to an exceptionally high binding energy resolution that is prevented in conventional XPS due to the strike of a balance between resolution and transmission energies for photoelectrons. Synchrotron radiation is also tunable over a wide wavelength range. Thus, an optimum X-ray photon energy can be selected, resulting in maximum photoionization cross-sections for probing a particular core level; non-destructive/non-invasive depth profiling can be achieved simultaneously. Most of the time, chemisorbed oxygen species are mistakenly taken as H_2_O, ${\mathrm{OH}}^{-}$, or lattice O^2−^ ion components due to complexations in the measurement system accompanied by a lack of interest in the chemisorbed oxygen ions. As a result, the final narrative may lead the sensor designer to an assessment of the sensing mechanism in a different way than it actually is. XPS bridges the well-established quantification in addition to the chemical state resolution to identify the surface stoichiometry with an understanding of the oxygen species on the surface, which are detrimental to chemical sensing. Electron binding energies (BE) specified for the O 1s main photoelectron line using synchrotron-based XPS for determining the amount and position of the adsorbed-chemisorbed oxygen ions can be applied in the laboratory-based XSP peak fitting and deconvolution procedures. This will bring accurate fitting/deconvolution for binding energy values for the absorbed chemisorbed oxygen ions.

## 4. Experimental

During photoemission experiments, varying photon energies were used to increase the surface sensitivity and efficiency for the main photoelectron lines by obtaining a suitable amount of kinetic energy (KE) for the photoelectrons. W 4f and O 1s were referenced to the Fermi level. Gold (Au) and carbon (C) corrections were also applied. The analysis chamber pressure was maintained at 5 × 10^−10^ mbar. The deconvolution analysis was completed with a Shirley background and Gauss–Lorentzian sum functions. The WO_3_ thin films were grown to 200 nm in thickness at 700 °C via metalorganic chemical vapor deposition (MOCVD). The details of the growth process can be found in the reference [98]. The X-ray photon energies of 70, 100, 300, 600, and 1000 eV were used for O 1s and W 4f photoelectron excitations. The balance band analysis was completed with 50, 100, 200, and 300 eV X-ray photons. During experiments, different electron analyzer sample surface normal angles were utilized to alter the surface sensitivity as required. The gas-sensing experiments were conducted with an H_2_ concentration ranging from 1000 to 4000 ppm balanced with N_2_ at 100 °C, 250 °C, and 400 °C. Measurements of 20, 5, and 1 min pulses were used for H_2_ exposure. During the isothermal hold, these pulses were balanced with pure N_2_ (with 1% O_2_ background). Sensor response (S) is defined as presented in Equation (1), where R_Air_ is the resistance measured without gas exposure, and R_H_ is the resistance measured under H_2_ exposure. If the resistance change is negative, then the sensor response (S) will be specified as an “n-type response”. The absolute maximum of the electrical resistance change is phrased as **“S_max_”. “S_max_”** represents the maximum change in the sensor response (sensitivity). The sensors were prepared with platinum (Pt) interdigitated electrodes (IDEs) due to their long-term chemical and electrical stability [7,8,99]. The temperature sensor and heating elements are also integrated into the gas sensor architecture.

(1) $$S=Sensor\;Response\equiv \left(Sensitivity\right)=\left(\frac{R_{Air}}{R_{Hydrogen}}\right)$$

## 5. Investigation of Adsorbed Oxygen Ions, Surface Chemistry-Homogeneity, and Work Function (Φ) of Semiconducting Metal Oxides (SMOs)

The quantity of oxygen molecules taken up by SMOs depends on temperature, oxygen partial pressure, and surface properties of SMOs. Dissociative adsorption of oxygen, chemisorption, and the continuous replenishment on the surface during sensing (Red-Ox) reactions are prerequisites for chemical sensing. Some of the best surface activity indicators are measuring work function (Φ) and oxidation state variations. Chemical homogeneity on the surface is determined by the purity of the sensing material and the distribution and concentration of different oxidation states on the sensing material SMO surface (such as distribution and concentration of W^6+^, W^5+^, and W^4+^ on a WO_3_ surface). This distribution is a significant factor in the interaction between the target gas and the sensor surface and in the interaction with chemisorbed $(O_{2\left(\mathrm{ads}\right)}^{-},{\;O}_{2\left(\mathrm{ads}\right)}^{2-},\;O_{\left(\mathrm{ads}\right)\;}^{-},{\;O}_{\left(\mathrm{ads}\right)}^{2-}$) ions. Quantifying chemisorbed oxygen ions and the correlation with the sensing mechanism and the sensitivity remains challenging. We explored the amount of chemisorbed oxygen ions, work function (Φ), surface stoichiometry, and homogeneity of WO_3_ as a function of temperature from 25 to 400 °C under different O_2_ and H_2_ pressures.

### 5.1. Surface Topography of WO_3_ through LEEM

**Figure 3:**
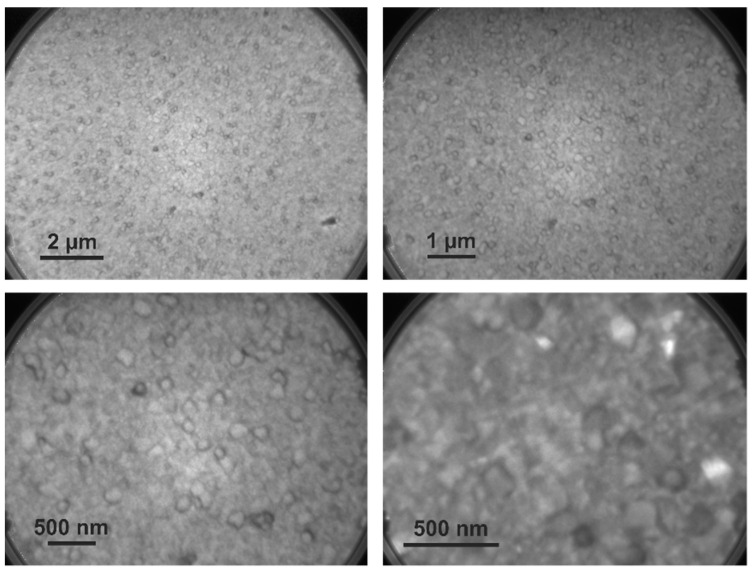
LEEM images of the WO_3_ surface with different magnifications; images were collected with 5 eV electrons.

LEEM provides imaging of surface topography to identify structural domains such as those formed in polycrystalline oxide thin films. In LEEM, work function (Φ) variation over the surface determines the image contrast. In turn, surface topography, grain boundaries, porosity, and stoichiometry affect the work function (Φ), thus leading to different visual validation of the surface topography compared to SEM. LEEM uses the reflection of a beam of low-energy electrons to create an image of a surface with very high lateral resolution. The short inelastic mean free path of electrons restricts probing depth to the uppermost atomic layer in LEEM. The bright-field image of the WO_3_ thin film is shown in Figure 3 in three different magnifications. The topographic description of the WO_3_ surface is the formation of a continuous thin film with a homogeneous grain arrangement with a few protruding abnormal growth grains featuring a higher surface roughness. Several different domains are visible. Higher magnification images suggested that a WO_3_ thin film nanostructure contains potential differences in height and chemically modified work function (Φ) differences. The dark-looking areas around the grain boundaries visible throughout the surface represent the lower work function (Φ) domains, which are axiomatically attributed to the lower oxidation state. XPEEM analysis was completed for further understanding of this.

### 5.2. Mapping Oxidation State Homogeneity on the WO_3_ Surface via XPEEM

It is well known that any surface may possess laterally altering chemical and/or physical features. The XPS spectrum only catches the average of all surfaces; in contrast, XPEEM is utilized in cases where spatially resolved chemical state information is required. XPEEM is a novel spectroscopic imaging technique that offers high lateral resolution regarding the spatial origin of the emitted electrons and leads to a 2D resolution of the chemical state on the surface. XPEEM exploits the characteristics of synchrotron radiation to implement a laterally resolved version of XPS. Regular XPS spectral analysis can capture the average chemistry, and XPEEM can chemically identify features down to 50 nm. This lateral resolution capability of XPEEM adds more sensitivity to the XPS. The image contrast in XPEEM arises from three primary contributors: surface topography, work function (Φ), and oxidation state (stoichiometry). In our measurements, we utilized high-energy photons, so it could be reasonable to claim that minute differences in the work function (Φ) will not affect the image contrast as opposed to the LEEM imaging. The image contrast will be the surface’s topographic and chemical ordering (oxidation state-stoichiometry) differences. The depth probed from the surface is ~1 nm.

**Figure 4:**
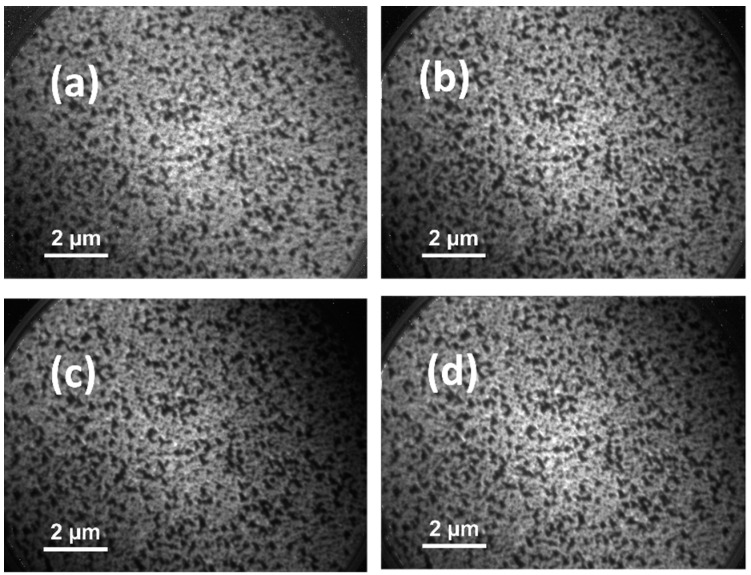
XPEEM images showing chemically selected scans of W 4 f. The images were recorded with a 12.4 µm field of view (FoV), corresponding to the diagonal distance in the images given. The X-ray excitation photon energies is increasing from 75 to 90 from (**a**–**d**) with a step size of 5 eV.

Figure 4a–d shows the high-resolution XPEEM chemically selective images of WO_3_ with four different electron kinetic energies to qualify the differentiation in the oxidation states of W^6+^, W^5+^, and W^4+^ from grain-to-grain resolution. Electron kinetic energies were altered as a step size of 5 eV by adjusting the X-ray excitation photon energies from 75 to 90 eV; this way, if there was any heterogeneous accumulation of the above-mentioned oxidation states (W^6+^, W^5+^, and W^4+^ +), it would be revealed in the XPEEM images. During XPEEM analysis, the same exact physical location on the WO_3_ thin film surface was used during imaging to eliminate the topological differences’ effects on XPEEM images. The images that are given in Figure 4 state that inhomogeneity in the oxidation state in the 50 nm range does not exist throughout the WO_3_ thin film. W^6+^, W^5+^, and W^4+^ oxidation states are homogenously distributed on the grain size level through the 12.4 µm FoV images. The homogenous distribution of W^6+^, W^5+^, and W^4+^ oxidation states is vital due to the catalytic effect of single- or double-charged oxygen vacancies ($V_{O}^{•},V_{O}^{••}$) resulting from W^5+^ and W^4+^ oxidation states. The finding is significant for sensor designers to optimize surface properties, such as future demands for an inexpensive integration of catalytic effects on SMO-type sensors via surface defect engineering. In each XPEEM image, surface contrast is dictated by the surface topography and oxidation state. The lighter-looking areas represent trench-hole like structures, while the darker-looking regions show protruding features. It is evident that the distribution of the oxidation states of W^6+^, W^5+^, and W^4+^ is homogeneous; their amount needs to be clarified by high-resolution XPS analysis, which has been completed with the results presented in Section 5.4.

### 5.3. Work Function (Φ) Measurements

**Figure 5:**
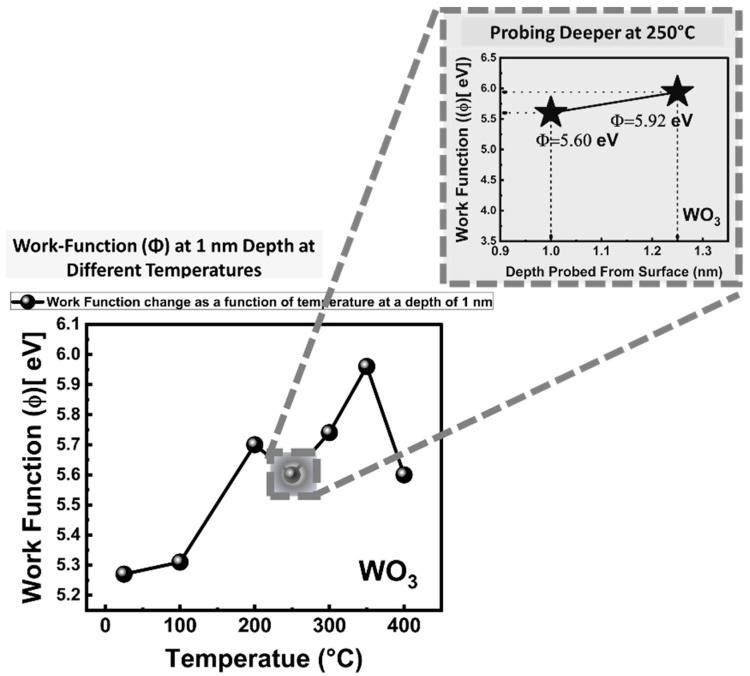
WO_3_ thin film work function (Φ) measurements from 25 to 400 °C. The WO_3_ thin film is directly deposited over the Pt (platinum) interdigitated electrodes for gas sensor testing.

Work function (Φ) is the electronic charge that the energy barrier electrons must overcome when leaving the solid surface. Figure 5 details the work-function (Φ) measurements of the WO_3_ from 25 °C to 400 °C at the 1 nm depth, while the inset shows the change of Φ at in-depth measurement points at 250 °C. Work function (Φ) calculations were completed by fitting the Fermi level, then making linear the extrapolation of the secondary cut-off region to zero energy intensity, and subtracting the corresponding binding energy value from the excitation photon energy. The work function (Φ) continuously increased from 25 °C to 200 °C. The Φ values were 5.27 eV at 25 °C, and increased to 5.31 eV at 100 °C. At 200 °C and 250 °C, work function (Φ) values were 5.70 eV and 5.60 eV, respectively. The relative decrease at 250 °C compared to 200 °C can be explained based on the increase in the adsorbed oxygen ions concentration. At 300 °C, an increase in the work function was measured. This was attributed to both desorption of chemisorbed oxygen ions from the surface and the annihilation of the oxygen vacancy sites via the incorporation of the chemisorbed oxygen ions into the oxygen sublattice on the surface. At 350 °C, work function (Φ) increased up to the value of ~5.97 eV. The drastic decrease in the work function (Φ) came at 400 °C. At this temperature, a chemical reduction event occurred in WO_3_. Thus, a sudden decrease in the work function (Φ) down to 5.60 eV was observed. It is known that a perfect stoichiometry WO_3_ possesses a Φ close to ~6 eV [100]. Surface adsorbed oxygen plays a central but generally overlooked function in the samples work function (Φ). It is known that chemisorbed oxygen ions dominate chemical and physical properties at the imminent surface. Based on this, we changed the excitation X-ray photon energy to probe less deep of a zone at 250 °C. The increase in the Φ from 5.60 eV to 5.92 eV and the depth probed changed from 1 nm to 1.25 nm at 250 °C indicates that the depth of 1.25 nm is the border region of the W^6+^ oxidation state that takes over the majority phase from W^5+^.

### 5.4. Amount of Adsorbed Oxygen Species on WO_3_ Sensor Surface

Understanding of metal oxide (SMOs) surface–oxygen interactions is essential for designing high-sensitivity gas sensors. The open literature needs to quantify chemisorbed oxygen species ($O_{2\left(\mathrm{ads}\right)}^{-},{\;O}_{\left(\mathrm{ads}\right),\;}^{-}O_{2\left(\mathrm{ads}\right)}^{2-},{\;O}_{\left(\mathrm{ads}\right)}^{2-}$) and oxygen vacancies ($V_{O}^{••}$) for SMOs utilized in gas sensing. Chemisorption and oxygen dissociation onto a perfectly stoichiometric SMO surface is not possible. Non-stoichiometric SMOs are far more efficient for the dissociative adsorption of oxygen and target gases (such as H_2_) [28]. We report synchrotron-based XPS measurements and a chemical quantification of WO_3_ in an as-deposited state and after oxygen (O_2_) and hydrogen (H_2_) exposures for determining the corresponding amounts of chemisorbed oxygen ions ($O_{2\left(\mathrm{ads}\right)}^{-},{\;O}_{\left(\mathrm{ads}\right),\;}^{-}O_{2\left(\mathrm{ads}\right)}^{2-},{\;O}_{\left(\mathrm{ads}\right)}^{2-}$), water/hydroxide groups (H_2_O/${\mathrm{OH}}^{-}$), and lattice oxygen ions ($O_{\mathrm{lattice}}^{2-}$). We also measured the amounts of different oxidation states of tungsten (W); W^6+^, W^5+^, and W^4+^, with the latter two, W^5+^ and W^4+^, being sub-stoichiometric forms of WO_3_, which are direct consequences of oxygen vacancies ($V_{O}^{•},{\;V}_{O}^{••}$). Equations (2), (14) and (15) show the formation of oxygen vacancies ($V_{O}^{•},{\;V}_{O}^{••}$), with W^5+^ and W^4+^ in Kroger–Vink notation. Oxygen vacancies on SMO surfaces promote the adsorption of oxygen molecules (O_2_) and further facilitate the formation of chemisorbed species given in Equations (3)–(6). It is vital to design such surfaces with active adsorption sites while keeping the overall stoichiometry high enough to keep semiconducting properties effective.

(2) $$O_{o}^{x}\;↔\;V_{o}^{••}+2e^{-}+\frac{1}{2}O_{2}$$

(3) $$O_{2\left(gas\right)}+e^{-}\to O_{2\;\left(adsorbed\right)}^{-}$$

(4) $$O_{2\left(gas\right)}+2e^{-}\to 2O_{\left(adsorbed\right)}^{-}$$

(5) $$O_{2\left(adsorbed\right)}^{-}+e^{-}\to O_{2\left(adsorbed\right)}^{2-}$$

(6) $$O_{2\left(adsorbed\right)}^{2-}\;\to 2\;O_{\left(adsorbed\right)}^{-}$$

**Figure 6:**
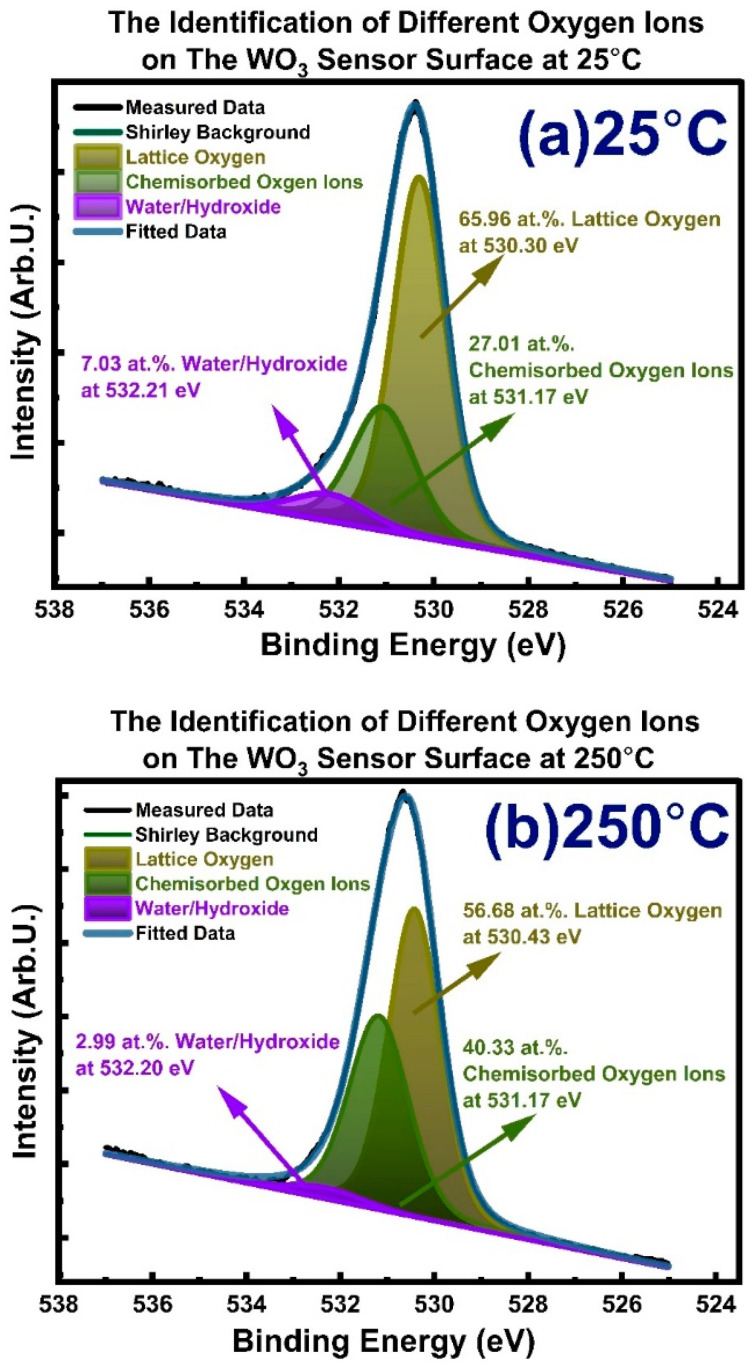
O 1s peak analysis from 1 nm depth on the WO_3_ sensor surface at two different temperatures. The XPS analysis shows amounts of different oxygen-ion-containing species in atomic percentages (at.%) at 25 °C (**a**) and at 250 °C (**b**).

We completed XPS analysis at a 1 nm depth from the sensor surface, which is the most relevant depth for the chemical gas sensing reactions. During heating to designated temperatures ranging from 25 °C to 400 °C, 10^−2^ mbar O_2_ gas exposures for 20 min were applied on WO_3_ samples to simulate the gas sensor operational/working conditions. Figure 6a,b shows the XPS spectrum of oxygen O 1s from WO_3_ at 25 °C and 250 °C temperatures, respectively. The quantification of different oxygen-ion-containing species, water/hydroxide groups (H_2_O/${\mathrm{OH}}^{-}$), chemisorbed oxygen ions ($O_{2\left(\mathrm{ads}\right)}^{-},{\;O}_{\left(\mathrm{ads}\right),\;}^{-}O_{2\left(\mathrm{ads}\right)}^{2-},{\;O}_{\left(\mathrm{ads}\right)}^{2-}$), and ordinary lattice oxygen ions ($O_{\mathrm{lattice}}^{2-}$) at 25 °C and 250 °C were completed through precise peak fitting-deconvolution analysis of the O 1s photoelectron spectrum. This method is unique, possesses surface sensitivity and differentiating capability, and is powered by the resolution power of the synchrotron. Thus, it provides precise binding energy analysis and quantification to shed light on the sensitivity and sensing mechanism correlation between temperature and surface adsorbed oxygen ions and hydrogen and oxygen interactions.

As seen in Figure 6a, at 25 °C, in the O 1s spectrum, binding energies (BE) are 532.21, 531.10, and 530.30 eV for water/hydroxide, chemisorbed oxygen, and ordinary lattice oxygen, respectively. The binding energy (BE) values were altered to higher energy as the temperature was raised to 250 °C, as seen in Figure 6b: 532.20, 531.17, and 530.43 eV. The binding energies (BE) reported here for water/hydroxide, chemisorbed oxygen, and ordinary lattice oxygen are in good agreement with the literature values reported from different semiconducting metal oxides (SMOs) [28]; at the same time, it is hardly difficult to find literature that explicitly reports the binding energy (BE) values for chemisorbed oxygen ions. The corresponding amounts of the oxygen ions found in water/hydroxide groups (H_2_O/${\mathrm{OH}}^{-}$), chemisorbed oxygen ions ($O_{2\left(\mathrm{ads}\right)}^{-},{\;O}_{\left(\mathrm{ads}\right),\;}^{-}O_{2\left(\mathrm{ads}\right)}^{2-},{\;O}_{\left(\mathrm{ads}\right)}^{2-}$), and ordinary lattice oxygen ions ($O_{\mathrm{lattice}}^{2-}$) at 25 °C and 250 °C are provided in Figure 6a,b, respectively. There is a significant increase in the chemisorbed oxygen ions once the WO_3_ is heated from 25 °C to 250 °C under conditions similar to the gas-testing conditions. The amount of chemisorbed oxygen ions increased from 27.01 at.%. to 40.33 at.%. The decrease in the amount of lattice oxygen ions is related to the significant increase in the chemisorbed oxygen ions, which decreases the X-rays reaching the lattice oxygen region staying under the chemisorbed oxygen ions.

**Figure 7:**
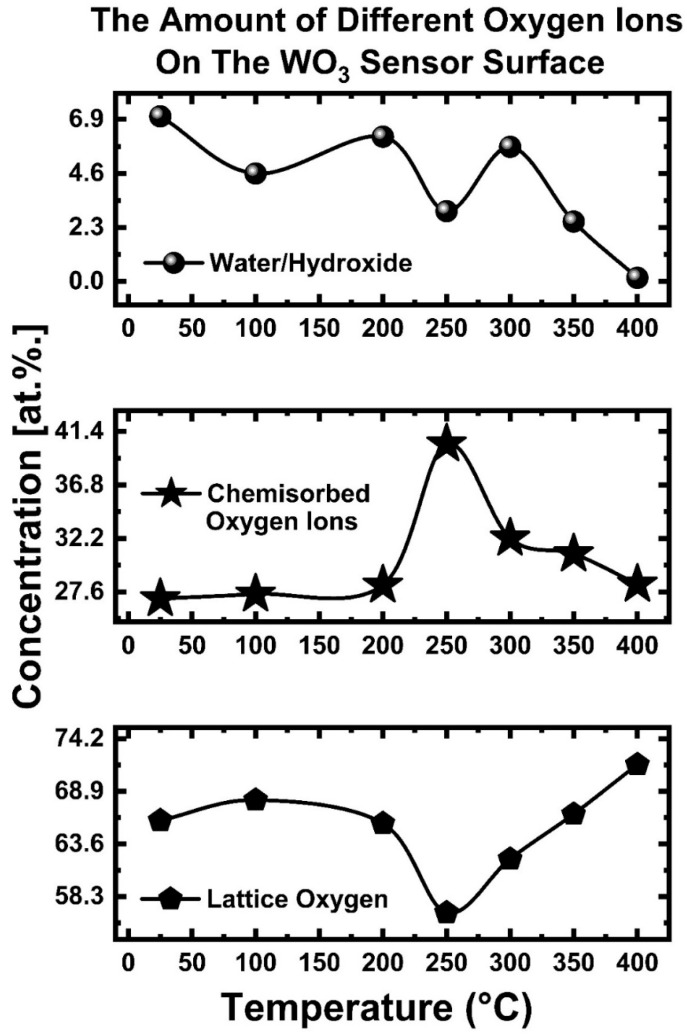
Quantification of different adsorbed oxygen ions on the WO_3_ gas sensor surface as a function of temperature from 25 °C to 400 °C. The analysis was completed within data acquisition of 1 nm depth from the WO_3_ gas sensor surface. From top to bottom: oxygen ions in water/hydroxide groups, chemisorbed oxygen ions, and lattice-oxygen-occupied concentrations were given.

Figure 7 shows and tabulates the amounts of water/hydroxide (H_2_O/${\mathrm{OH}}^{-}$), chemisorbed oxygen ions ($O_{2\left(\mathrm{ads}\right)}^{-},{\;O}_{\left(\mathrm{ads}\right),\;}^{-}O_{2\left(\mathrm{ads}\right)}^{2-},{\;O}_{\left(\mathrm{ads}\right)}^{2-}$), and lattice oxygen ions ($O_{\mathrm{lattice}}^{2-}$) measured on the WO_3_ gas sensor surface at temperatures changing stepwise from 25 °C up to 400 °C. As seen in Figure 7, the amount of chemisorbed oxygen ions at 25 °C is 27.01 at.%., while the amount of water/hydroxide (H_2_O/${\mathrm{OH}}^{-}$) is 7.03 at.%. The increase in temperature to 100 °C and then 200 °C did not change the amount of the water/hydroxide as well as chemisorbed oxygen species any more than 1 at.%., while the drastic change came at 250 °C. The measurements at 250 °C showed a drastic decrease in the amount of the water/hydroxide while showing a significant increase in the amount of the chemisorbed oxygen ions.

The maximum amount of the chemisorbed oxygen ions was detected at 250 °C, which amounts to 40.33 at.%. at a depth of 1 nm. As the temperature was further increased to 300 °C, the amount of chemisorbed oxygen ions drastically decreased down to 32.21 at.%. Analysis at 350 °C and 400 °C showed that the amount of chemisorbed oxygen ions decreased to 30.86 at.%. and 28.26 at.%, respectively. The thermal movement of the adsorbed species (water/hydroxide (H_2_O/${\mathrm{OH}}^{-}$) and chemisorbed oxygen ions ($O_{2\left(\mathrm{ads}\right)}^{-},{\;O}_{\left(\mathrm{ads}\right),\;}^{-}O_{2\left(\mathrm{ads}\right)}^{2-},{\;O}_{\left(\mathrm{ads}\right)}^{2-}$) was boosted with an increase in temperature, leading to fast desorption from the WO_3_ surface. We observed dissociative adsorption of oxygen ions (chemisorbed oxygen ions) maximized at the temperature of 250 °C. This was accompanied by a decrease in the amount of the lattice-occupied oxygen ions due to the physical blocking of the photoelectrons by the high concentration of chemisorbed oxygen ions.

Beyond the 300 °C temperature regime can be described as a dynamic lattice active region for an anionic defect-rich WO_3_ thin film. The dynamic lattice ($O_{\left[\mathrm{Dynamic}\;\mathrm{Lattice}\right]}^{2-}$) was previously introduced in Figure 2. Dissociative adsorption of oxygen gas is fast due to the high and well-distributed concentration of electron-rich W^5+^ and W^4+^ oxidation states and associated oxygen vacancies ($V_{o}^{•}{\;\mathrm{and}\;V}_{o}^{••}$) on the WO_3_ surface. A fast and reversible incorporation of chemisorbed oxygen ions into the oxygen sub-lattice in the defective WO_3_ is described in Equations (7)–(9). The equations given below show the incorporation of $O^{2-}$ and $O^{-}$ into the WO_3_ lattice in a reversible fashion facilitated by the single- ($V_{o}^{•}$) and double-charged ($V_{o}^{••}$) oxygen vacancies.

(7) $$3V_{o}^{•}+e^{-}+O^{2-}\;↔\;3O_{o\;\left[Dynamic\;Lattice\right]}^{x}$$

(8) $$V_{o}^{•}\;+O^{-}\;\;\;\;\;↔\;O_{o\;\left[Dynamic\;Lattice\right]}^{x}$$

(9) $$V_{o}^{••}+O^{2-}\;\;\;↔\;O_{o\;\;\left[Dynamic\;Lattice\right]}^{x}$$

**Figure 8:**
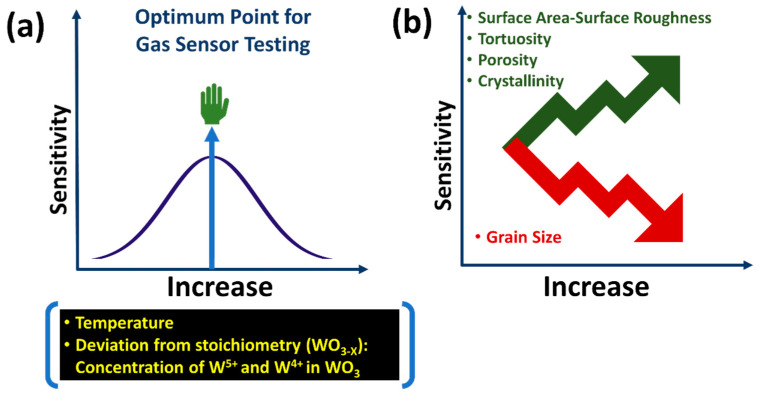
Some of the critical trends in gas sensors are shown above. (**a**) An increase in temperature first will increase the sensitivity due to a rise in the amount of the chemisorbed oxygen ions. After a specific threshold value passes, the sensitivity (S) decreases rapidly due to the termination of chemisorbed oxygen ions and the stoichiometry of the metal oxides (SMOs) deviates due to a chemical reduction event. (**b**) Other parameters will have a linear effect on the sensitivity.

The dependence of the sensitivity (the magnitude of the gas sensor response) of SMOs’ gas sensors on temperature, deviation from stoichiometry (WO_3-x_), surface area, surface roughness, tortuosity, porosity, and crystallinity is given in Figure 8a,b. We showed that the amount of the chemisorbed oxygen ions ($O_{2\left(\mathrm{ads}\right)}^{-},{\;O}_{\left(\mathrm{ads}\right),\;}^{-}O_{2\left(\mathrm{ads}\right)}^{2-},{\;O}_{\left(\mathrm{ads}\right)}^{2-}$) follows a curve-like pattern that reaches a peak around 250 °C (see Figure 7). After that threshold, the amount of chemisorbed oxygen ions decreases rapidly with a further temperature increase. The same trend will be valid for the temperature due to its destructive effect on stoichiometry and the amount and stability of the chemisorbed oxygen ions. The sensor testing temperature strongly affects gas diffusion through tortuosity in the sensing layer, thus relatively increasing the surface area contacted by the target gas and increasing the sensitivity. However, equally necessary competing factors have opposing temperature dependences, and their dominance dictates the suitable testing temperature for the gas sensors. The threshold points for temperatures and the deviation from the SMOs’ stoichiometry will depend on the choice of sensing material SMO (such as but not limited to WO_3_, SnO_2_, TiO_2_, MoO_3_, ZnO, and In_2_O_3_). Based on the measurements presented in Figure 7, we proposed the trends for temperature and deviation from stoichiometry (WO_3-x_) in Figure 8a for chemical gas sensing. The sensitivity is a Gaussian-like function of the temperature and the deviation from the SMOs’ stoichiometry (amount of W^4+^ and W^5+^ in WO_3_) (see Figure 8a). However, the sensitivity exhibits a linear-like relationship with porosity, tortuosity, crystallinity, and grain size.

Semiconducting metal-oxide-based (SMO) sensors exhibit a maximum sensor response (sensitivity) at a particular optimum temperature. The amount of chemisorbed oxygen ions and deviation from stoichiometry dictate this temperature dependency, as illustrated in Figure 8a. A further increase in temperature beyond this “sweet point” will decrease the sensitivity due to the undesired desorption of chemisorbed oxygen ions ($O_{2\left(\mathrm{ads}\right)}^{-},{\;O}_{\left(\mathrm{ads}\right),\;}^{-}O_{2\left(\mathrm{ads}\right)}^{2-},{\;O}_{\left(\mathrm{ads}\right)}^{2-}$). The maximum amount of the chemisorbed oxygen ions will lead to the maximum amount of electrons being released, leading to maximal gas sensor response (sensitivity).

A near-perfect stoichiometric WO_3_, consisting of a high W^6+^, will not result in a desired sensitivity. It is possible to obtain an order-of-magnitude-higher sensitivity by using WO_3_ containing W^5+^ and W^4+^, but this will be self-limiting. Beyond “a critical point-sweet point”, the amount of the non-stoichiometric phase will have an adverse effect on the sensitivity. The deviation from stoichiometry, or in other words, the existence of the W^5+^ and W^4+^ oxidation states for the current case of WO_3_, is a direct consequence of oxygen vacancies ($V_{O}^{•}\;\mathrm{and}\;V_{O}^{••}$) as introduced in the following Kroger–Vink defect equations: Equations (2), (7)–(9), (14) and (15). These electron-rich, sub-stochiometric W^5+^ and W^4+^ oxidation states are facilitators of the oxygen gas’ dissociative adsorption (chemisorption) as well as of target gases such as H_2_. The further increase from a “sweet point” in the non-stoichiometry (concentrations of W^5+^ and W^4+^) in the sensing SMOs will destroy the sensor response sensitivity due to chemical reduction of the SMOs stoichiometry. This destruction of sensor response is due to the formation of an intolerable amount of oxygen vacancies ($V_{O}^{•}\;\mathrm{and}\;V_{O}^{••})$, as they will transform the WO_3_ or any other SMO surfaces with semiconducting behavior to a metallic conductor, thus terminating or diminishing the sensor response sensitivity.

**Figure 9:**
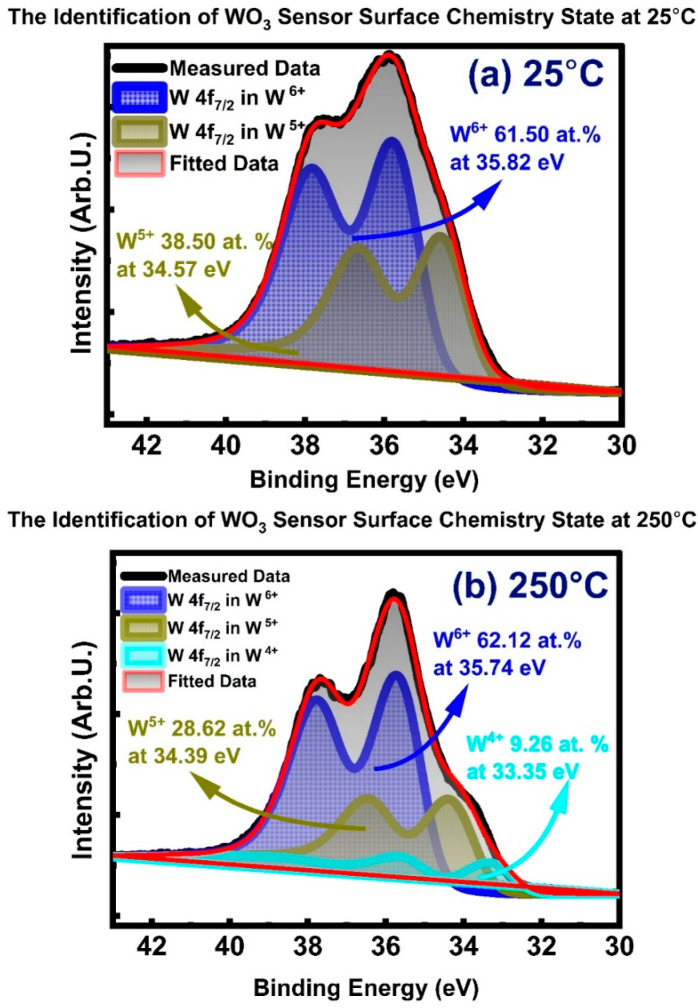
Chemical phase identification of WO_3_ sensor surface within a 1 nm depth from the WO_3_ sensor surface by XPS W 4 f peak analysis at 25 °C (**a**) and 250 °C (**b**).

Figure 9 gives the quantitative oxidation state breakdown of WO_3_ at the surface represented by a 1 nm analysis depth at 25 °C (a) and 250 °C (b). The results indicate that the WO_3_ surface is built of W^5+^ and W^6+^ at 25 °C. In addition to W^5+^ and W^6+^, newly formed W^4+^ was detected at 250 °C. The corresponding number of chemical states and binding energies is provided in Figure 9. Increasing the temperature from 25 to 250 °C did not change the amount of the W^6+^ drastically, while it significantly converted the W^5+^ to W^4+^. After exposure to 250 °C, some portion of the W^5+^ reduced to W^4+^, resulting in a shoulder formation on the lower binding energy side, as can be seen in Figure 9b. The presence of the W^4+^ can be expressed in Kroger–Vink notation as presented in Equations (14) and (15). The reduction of W^5+^ to W^4+^ results from the singly charged oxygen vacancies ($V_{o}^{•}$) forming doubly charged oxygen vacancies ($V_{o}^{••}$), as seen in Equation (15).

The W^5+^ concentration decreased from 38.50 at.% to 28.62 at.%. as the temperature was raised from 25 °C to 250 °C. At 250 °C, the WO_3_ surface was composed of 62.12 at.% W^6+^, 28.62 at.% W^5+^, and 9.26 at.% W^4+^. The binding energy (BE) values at 25 °C for W 4f_7/2_ from W^6+^ and W^5+^ oxidation states were 35.82 eV and 34.57 eV, respectively. The binding energy values for W^6+^ and W^5+^ oxidation states showed a very minor change at the temperature when raised to 250 °C; measured binding energy values were 35.74 eV and 34.39 eV for W^6+^ and W^5+^ oxidation states, respectively. The binding energy values reported here are in good agreement with literature reported values [4].

In comparison to the results measured within a 2 nm depth from the surface (published elsewhere [98]), an amount of the W^5+^ oxidation state was detected more strongly at a 1 nm depth from the sensor surface; this implies that more surface sensitivity brings a deeper understanding of the gas sensor surface, as a 1 nm depth represents the most relevant sensing activity depth from the surface. The W^4+^ phase appeared at 250 °C with a binding energy of 33.35 eV. The W^4+^ and W^5+^ oxidation states are the catalytically active sites for dissociative oxygen adsorption. The W^4+^ and W^5+^ also help the H_2_ dissociation and, further, the oxygen vacancy ($V_{o}^{•},\;V_{o}^{••}$) facilitated the oxidation reactions shown in Equations (10)–(13) and (16).

**Figure 10:**
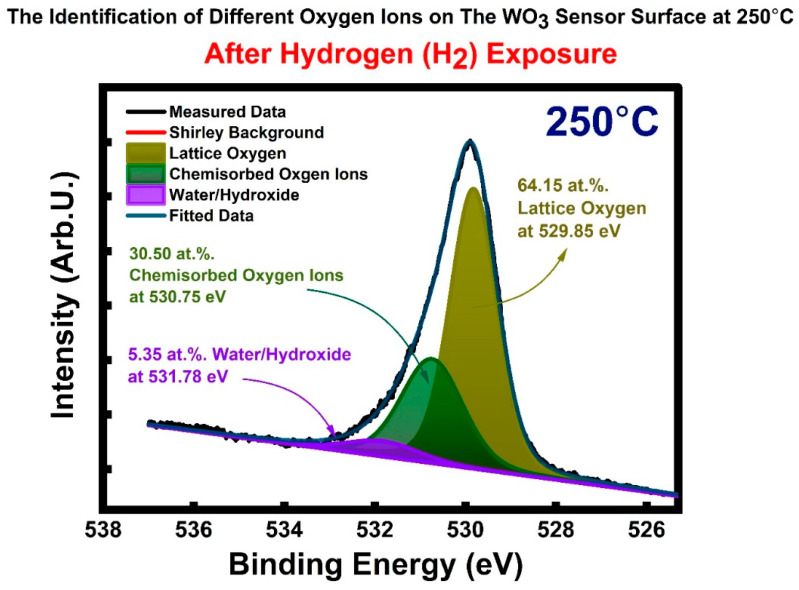
Oxygen O 1s peak analysis at 250 °C at a 1 nm depth on the WO_3_ sensor surface after H_2_ exposure.

Before proceeding with H_2_ sensor testing, the WO_3_ sample was exposed to the H_2_ in the XPS chamber for 20 min at 250 °C. The measurement of the O 1s photoelectron line and corresponding amount of chemisorbed oxygen ions ($O_{2\left(\mathrm{ads}\right)}^{-},{\;O}_{\left(\mathrm{ads}\right),\;}^{-}O_{2\left(\mathrm{ads}\right)}^{2-},{\;O}_{\left(\mathrm{ads}\right)}^{2-}$) of water/hydroxide (H_2_O/${\mathrm{OH}}^{-}$) and lattice-related oxygen ions ($O_{\mathrm{lattice}}^{2-}$) are calculated based on the deconvolution analysis shown in Figure 10. The binding energy values for each species shifted ~0.5 eV toward a lower binding energy compared to the WO_3_ sensor not exposed to the H_2_. The binding energy (BE) values are 531.78 eV for water/hydroxide, 530.75 eV for chemisorbed oxygen ions, and 529.85 eV for ordinary lattice oxygen ions. The amount of the chemisorbed oxygen ions at 250 °C without H_2_ exposure was 40.33 at.% (see Figure 6b). The amount of chemisorbed oxygen ions decreased to 30.50 at.% after H_2_ exposure at 250 °C; this was accompanied by an increase in the number of water/hydroxide groups, as seen in Figure 10. The increase in water/hydroxide groups was expected since the chemical reaction between H_2_ and chemisorbed oxygen ions leads to H_2_O formation (see Equations (10)–(16)). The amount of water/hydroxide groups was 2.8 at.% without H_2_ exposure, as seen in Figure 6b, while after H_2_ exposure, this amount increased to 5.35 at.%, as seen in Figure 10. Lattice-related oxygen ions concentration was 56.68 at.% before H_2_ exposure, while after H_2_ exposure, the amount increased to 64.15 at.%. This increase is associated with consumption of the chemisorbed oxygen ion by the WO_3_ surface with H_2_ exposure, which in turn physically opens up the way for more lattice-related core oxygen ions related to electron detection in the electron analyzer.

## 6. H_2_ Sensor Testing at 250 °C

**Figure 11:**
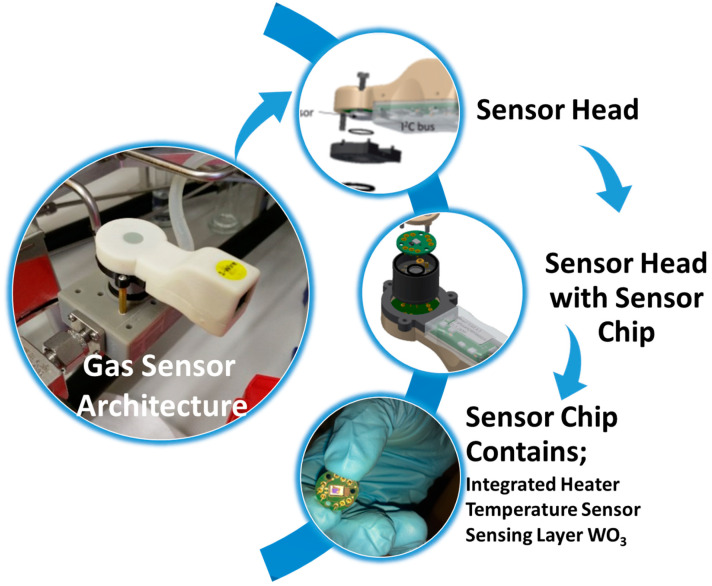
Sensors (white) soldered into circuit boards (green) for insertion into the mobile gas measuring station.

Considering the maximum amount of the chemisorbed oxygen ions and work function (Φ) value, we decided on testing at 250 °C. We completed gas sensor testing in the following one-wire bus configuration under 5 V. The Figure 11 shows the details of the sensor architecture used in the H_2_ testing. The sensor architecture included an integrated platinum heater and the temperature was monitored through an integrated Pt-1000 heat sensing element on the back of the gas sensing layer.

The elementary reactions regarding the chemical sensing in n-type semiconductors are given in Equations (10)–(16) for a reducing gas such as H_2_. The adsorption of oxygen consumes electrons, as seen in Equations (2)–(9). Subsequently, reducing gas, such as the given H_2_, counteracts the adsorbed oxygen ions through a process that extracts the chemisorbed oxygen ions from the surface and releases electrons back to the conduction band, decreasing the electrical resistance again. Hydrogen (H_2_) is a potent reducing agent and possesses electrophilic properties, leading to rapid dissociative-adsorption (Equations (10)–(12)) on the SMOs’ surfaces with defective qualities. This occurs such as in our case for WO_3_ with a high density of homogeneously distributed W^5+^ and W^4+^ defect sites. Oxygen molecules cannot be absorbed on fully oxidized SMO surface sites; conversely, the dissociation adsorption of oxygen happens at the oxygen vacancy ($V_{O}^{•},{\;V}_{O}^{••}$) sites (see Equation (2) and (7)–(9)). The oxygen vacancy sites are directly proportional to the amount of the reduced W^5+^ and W^+4^ phases due to the electrical neutrality of the host lattice.

**Figure 12:**
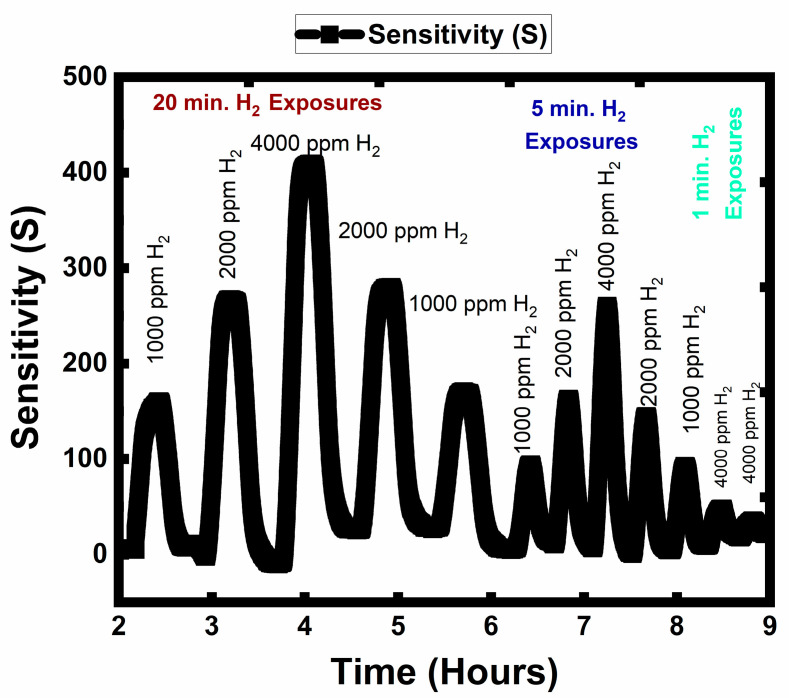
Dynamic sensor response of WO_3_ against H_2_ at 250 °C in the microsensor architecture.

Figure 12 shows the time-dependent dynamic resistance change curve for the WO_3_ at 250 °C. The sensor showed oxygen deficit n-type semiconducting metal oxide behavior against the reducing gas (H_2_). The S_max_ values were 167.2, 274.4, and 414.7 for the 20 min. pulses of 1000, 2000, and 4000 ppm of H_2_, respectively. The identical tendency was valid for the 5 min. pulses and the S_max_ values were 109.3, 174.6, and 272.4, respectively. For 1 min. pulses, the S_max_ were 63.8 and 51.3 for 4000 ppm of H_2_. The sensing of H_2_ on the WO_3_ surface was rapid even under 30 s exposures; the sensor was highly responsive. Additionally, it showed the capacity to distinguish between the different concentration levels of the target gas with proportionally changing sensor responses (sensitivity). The sensor’s high response towards H_2_ can be explained based on three distinct characteristics: (i) surface chemistry on catalytic activity towards H_2_ (sensing) and O_2_ (recovery) dissociation/adsorption, (ii) micro-sensor architecture, and (iii) a higher number of surface adsorption sites. It should be noted that catalytically active materials, such as platinum (Pt), gold (Au), or palladium (Pd), were not included on the WO_3_ surface. The WO_3_ thin film showed very high sensitivity and could distinguish different concentrations of H_2_ for different exposure times. The high sensitivity is directly related to the surface W^5+^ and W^4+^ oxidation states in WO_3_. As indicated in Equations (13) and (16)_,_ the H_2_ sensing is greatly facilitated and catalyzed by the W^5+^ and W^4+^ oxidation states.

As seen in Figure 9b, at 250 °C, the WO_3_ surface consists of W^4+^ (9.26 at.%), W^5+^ (28.62 at.%), and W^6+^ (62.12 at.%). W^4+^ is developed on the sensor surface based on transforming single-charge vacancies ($V_{o}^{•}$) to double-charged vacancies ($V_{o}^{••}$), as indicated in Equation (15). On the subject of a semiconducting metal oxide (SMOs) having high amounts of electron-rich centers, such as W^4+^ and W^5+^ ions, the reoxidation of the metal oxide through the adsorption and accommodation of O^2−^ ions into oxygen vacancy sites ($V_{o}^{•}$ and $V_{o}^{••}$) becomes very straightforward and instantaneous at temperatures as low as 250 °C. Supporting this explanation, sensor background drift in electrical resistance did not occur in our measurements. In other words, the WO_3_ surface interacted with H_2_ as a chemisorbed oxygen ion supplier for gas sensing reactions through the mechanisms shown in Equation (10) to Equations (13) and (16) and recovered to its initial electrical resistance by rapid oxidation once the gas flow of H_2_ was cut.

As seen in Figure 12, the recovery to the initial resistance after the H_2_ flow cut proves that there is a reversible consumption of chemisorbed oxygen ions. Post mortem XPS analysis also showed that, once the temperature reduced back to room temperature under 1% O_2_ background, the recovery of the WO_3_ sensor surface was observed through the reversible mechanisms presented in Equations (7)–(9), (14) and (15). The equations show the incorporation of O_2_ gas into $O_{\left[\mathrm{Dynamic}\;\mathrm{Lattice}\right]}^{2-}$ and $O_{\left[\mathrm{Dynamic}\;\mathrm{Lattice}\right]}^{-}$ (Equations (7)–(9)) and further into the WO_3_ lattice as the process facilitated by the single- ($V_{o}^{•}$) and double-charged ($V_{o}^{••}$) oxygen vacancies (Equations (14)–(16)).

(10) $$H_{2\left(gas\right)}+O_{\left(adsorbed\right)}^{2-}\;\to H_{2}O_{\left(desorbed\right)}+2e^{-}$$

(11) $$\frac{1}{2}H_{2\left(gas\right)}+O_{\left(adsorbed\right)}^{-}\;\to H_{2}O_{\left(desorbed\right)}+e^{-}$$

(12) $$H_{2\left(gas\right)}+O_{O\;\left(bulk\right)}^{X}\;\to H_{2}O_{\left(gas\right)}+V_{O}^{••}+2e^{-}$$

(13) $$\begin{array}{c} 2H_{2\left(gas\right)}+(2W^{5+}+V_{O}^{••}\overset{\begin{array}{c} adsoprtion\;and\;\\ dissociation\;on\;vacany\;site \end{array}}{↔}2O_{\left(adsorbed\right)}^{-})\\ \to 2W^{5+}+V_{O}^{••}+2H_{2}O_{\left(desorbed\right)}+2e^{-} \end{array}$$

(14) $$W_{W}^{X}+O_{O}^{X}\;↔\;V_{O}^{••}+\frac{1}{2}O_{2}+W_{W}^{\prime \prime }$$

(15) $$W_{W}^{\prime }+V_{O}^{\;•}\;↔\;V_{O}^{••}+W_{W}^{\prime \prime }$$

(16) $$\begin{array}{c} H_{2\left(gas\right)}+(W^{4+}+V_{O}^{••}\overset{\begin{array}{c} adsoprtion\;and\;\\ dissociation\;on\;vacany\;site \end{array}}{↔}O_{2\left(adsorbed\right)}^{-})\\ \;\to \;W^{4+}+V_{O}^{••}+H_{2}O_{\left(desorbed\right)}+2e^{-} \end{array}$$

## 7. Conclusions

We established novel applications of surface-sensitive techniques for understanding the semiconducting metal-oxide-based (SMO) sensing mechanism. We characterized the WO_3_ thin film’s oxidation state, crystallinity, morphology, and electrical and electronic properties. The correlation between gas sensor operation temperature, sensor responses, and the amount of the chemisorbed oxygen ions was established. The surface chemistry and homogeneity, supported by spatially resolved insight into the thin film’s chemical, electronic, and electrical properties, were evaluated using synchrotron-based XPS-UPS, XPEEM, and LEEM. We distinguished the chemisorbed oxygen ions ($O_{2\left(\mathrm{ads}\right)}^{-},{\;O}_{2\left(\mathrm{ads}\right)}^{2-},\;O_{\left(\mathrm{ads}\right)\;}^{-},{\;O}_{\left(\mathrm{ads}\right)}^{2-}$) from ordinary lattice ($O_{\mathrm{lattice}}^{2-}$) and water/hydroxide groups (H_2_O/${\mathrm{OH}}^{-}$) using precise synchrotron-based XPS measurements (see Figure 7). We quantified the amounts of chemisorbed oxygen, water/hydroxide, ordinary lattice oxygen ions, and the different oxidation states of tungsten (W) in tungsten oxide (WO_3_) as a function of temperature between 25–400 °C under H_2_ and O_2_ exposures. We observed an optimum temperature range for maximizing the chemisorbed oxygen ions’ concentration; in turn, we observed that the chemisorbed oxygen ions’ concentration dictates the maximum sensitivity that the SMO gas sensors can reach. The sensitivity’s relationship with the gas-testing temperature is dictated by the amount of chemisorbed concentration; the deviation from stoichiometry (in other words, the amount of W^4+^ and W^5+^ in the current case) is a complex form and follows a Gaussian trend and reaches a peak value followed by a rapid decline.

The gas-sensing reactions occur and continue by the consumption and replenishment of chemisorbed oxygen ions via surface reduction and oxidation (Redox) reactions. Chemisorbed oxygen ions, which hold the most critical part in the sensing mechanism of SMO-based gas sensors, were critically discussed, and comprehensive literature regarding their characterization was also included. The characterization methods relevant to the semiconducting metal-oxide-based (SMO) sensor analyses were reviewed, and their strengths and weaknesses were tabulated along with the physical and chemical data that can be extracted from each analytic, spectroscopic, and microscopic technique.

Gas sensor tests were carried out at 250 °C for hydrogen (H_2_) without adding expensive, catalytically active precious metals. The gas sensor tests were performed within a mobile microsensing architecture operating on a one-wire bus connection at 5 volts (V). The WO_3_ gas sensor showed an exceptionally high response (424) (S_max_) for H_2_ at 250 °C; this response was characterized based on surface defect engineering, including oxygen vacancies, chemisorbed oxygen ions, and surface stoichiometry of WO_3_. The WO_3_ sensor also showed outstanding repeatability upon multiple H_2_ exposures without degradation and drift.

The exceptionally high sensing characteristic is due to oxygen gas rapidly adsorbing on single- ($V_{o}^{•}$) and double-charged ($V_{o}^{••}$) oxygen vacancy defect sites created by the presence of W^5+^ and W^4+^ oxidation states. The XPS analysis confirmed the coexistence of W^4+^, W^5+^, and W^6+^ oxidation states in the WO_3_ sensor. The distribution of those oxidation states was laterally and vertically well-distributed and homogeneous, confirmed via XPEEM and UPS. These point defects increase surface adsorption sites and facilitate the chemisorption of oxygen and dissociative adsorption of hydrogen (H_2_), leading to a higher sensor response (S_max_). The finding is significant for sensor designers to optimize surface properties. Future sensor development dictates an inexpensive integration of catalytic effects into SMO-type sensors via surface defect engineering, the integration of surface vacancy sites ($V_{o}^{••}$ and $V_{o}^{•}$), and through the formation of lower oxidation states (W^5+^ and W^4+^) on the sensing material surface.

## Figures and Tables

**Table 1:** Basic and advanced analytic, microscopic, and spectroscopic techniques provide insights into the fundamental understanding of chemical gas sensors available for sensor designers.

Technique	Elemental Identification	Chemical State	Structure	Surface Defects	Bulk Defects	Morphology	Imaging	Depth Probed (nm)	Lateral Resolution (µm)	Quantification	In-Situ Applicability	Chemical State Mapping	Elemental Mapping	Electronic Properties
**(GI-)XRD**	**✓/X**	**✓/X**	**✓**	**✓(GI)**	**✓**	**X**	**✓**	**10-Bulk**	**≥10000**	**✓**	**✓**	**✓/X**	**✓/X**	**X**
**EXAFS**	**✓**	**X**	**✓**	**X**	**X**	**X**	**X**	**≥1000**	**>1000**	**✓/X**	**✓**	**X**	**X**	**✓**
**SEXAFS**	**✓**	**X**	**✓**	**X**	**X**	**X**	**X**	**1–10**	**>1000**	**✓/X**	**✓**	**X**	**X**	**✓**
**NEXAFS**	**✓**	**✓/X**	**✓**	**X**	**X**	**X**	**X**	**1–10**	**>1000**	**✓/X**	**✓**	**X**	**X**	**✓**
**XPS**	**✓**	**✓**	**X**	**✓**	✓*	**X**	**✓**	**0.5–10**	**50–100**	**✓**	**✓**	**✓**	**✓**	**✓**
**NAP-XPS**	**✓**	**✓**	**X**	**✓**	✓*	**X**	**✓**	**0.5–10**	**50–100**	**✓**	**✓**	**✓**	**✓**	**✓**
**UPS**	**X**	**✓**	**X**	**✓**	**X**	**X**	**X**	**0.5–5**	**150**	**✓**	**X**	**X**	**X**	**✓**
**LEED**	**X**	**X**	**✓**	**✓**	**X**	**X**	**✓**	**1–5**	**<0.1**	**X**	**✓**	**X**	**X**	**X**
**AES**	**✓**	**✓/X**	**X**	**✓**	✓*	**X**	**✓**	**0.5–10**	**<0.1**	**X**	**✓**	**X**	**✓**	**X**
**ISS/RBS**	**✓**	**✓/X**	**✓**	**X**	**X**	**X**	**X**	**0.3–3**	**150**	**✓**	**X**	**X**	**X**	**X**
**FT-IR**	**✓**	**✓/X**	**X**	**X**	**✓**	**X**	**✓**	**≥1000**	**≥5000**	**✓/X**	**✓**	**X**	**X**	**X**
**Raman**	**✓**	**✓/X**	**✓**	**X**	**✓**	**X**	**✓**	**≥1000**	**1–10**	**✓/X**	**✓**	**X**	**X**	**X**
**NMR**	**X**	**✓**	**✓**	**X**	**✓**	**X**	**X**	**>10^4^**	**>1000**	**✓**	**✓**	**X**	**X**	**X**
**LEEM**	**X**	**X**	**X**	**X**	**X**	**✓**	**✓**	**1–3**	**>1000**	**X**	**X**	**X**	**X**	**✓/X**
**XPEEM**	**✓**	**✓**	**X**	**✓**	✓*	**✓**	**✓**	**1–10**	**<0.1**	**✓**	**✓**	**✓**	**✓**	**✓**
**UV-VIS**	**X**	**X**	**X**	**X**	**✓**	**X**	**X**	**~1000**	**5–100**	**✓**	**X**	**X**	**X**	**✓**
**CL/PL**	**X**	**✓**	**X**	**✓/X**	**✓**	**X**	**✓**	**10–1000**	**≥1**	**✓/X**	**X/✓**	**✓**	**X**	**✓**
**XRF**	**✓**	**X**	**X**	**X**	**X**	**X**	**✓**	**≥1000**	**1000**	**✓**	**✓**	**X**	**✓**	**X**
**EDS-SEM**	**✓**	**X**	**X**	**X**	**X**	**✓**	**✓**	**>100**	**0.5**	**✓**	**✓/X**	**X**	**✓**	**X**
**H/R-EELS**	**✓**	**✓**	**X**	**✓**	**X**	**X**	**X**	**2–20**	**<0.1**	**✓/X**	**✓/X**	**X**	**✓/X**	**✓**
**TP(X)**	**X**	**✓/X**	**X**	**✓**	**X**	**X**	**X**	**>10^4^**	**>1000**	**✓**	**✓**	**X**	**X**	**X**

**✓**: Possible; X: Not possible; **✓/X**: Possible under special conditions with indirect, difficult, cumbersome processing, and substantial estimations without standards. ✓* With ion etching, such Argon etching depth profiling.

## Abbreviations

EXAFS	Extended X-Ray Absorption Fine Structure
SEXAFS	Surface Extended X-Ray Absorption Fine Structure
NEXAFS:	Near Edge X-Ray Absorption Fine Structure
XPS	X-Ray Photoelectron Spectroscopy
NAP-XPS	Near Ambient Pressure X-Ray Photoelectron Spectroscopy
UPS	Ultraviolet Photoelectron Spectroscopy
LEED	Low-Energy Electron Diffraction
AES	Auger Electron Spectroscopy
HR/-EELS	High-Resolution/Electron Energy-Loss Spectroscopy
ISS/RBS	Ion Scattering-Rutherford Backscattering Spectroscopy
FT-IR and Raman	Fourier Transform Infrared and Raman Spectroscopy
NMR	Nuclear Magnetic Resonance
LEEM	Low-Energy Electron Microscopy
XPEEM	X-ray photoemission electron Microscopy
UV-VIS	Ultraviolet–Visible Light Spectroscopy
CL/PL	Cathodoluminescence-Photoluminescence
XRF	X-Ray Fluorescence
EDS	Energy-Dispersive X-Ray Spectroscopy
SEM	Scanning Electron Microscopy
(GI)-XRD	(Grazing Incidence-) X-Ray Diffraction
TP(X)	Temperature Programmed (X:Reduction-Oxidation)
